# A Critical Review on the Development and Utilization of Energy Systems in Uganda

**DOI:** 10.1155/2022/2599467

**Published:** 2022-08-06

**Authors:** Ocident Bongomin, Patrick Nziu

**Affiliations:** ^1^Department of Manufacturing, Textile and Industrial Engineering, School of Engineering, Moi University, Eldoret, Kenya; ^2^African Centre of Excellence II in Phytochemical, Textile and Renewable Energy (ACE II-PTRE), Moi University, Eldoret, Kenya

## Abstract

We live in a world that is completely dependent on energy; thus, humankind can no longer live without power. With electricity being the main form of energy today, this has increased the complexity of our life today. In Uganda, electricity generation is mainly through hydropower, which puts the country in the bottleneck of overdependence on one source of energy. There are many energy systems out there that the country can use to diversify its electricity generation. Therefore, the need to understand the level of development and utilization of various energy systems has been the underlying question for this present study. A comprehensive literature survey was conducted using electronic databases, including ScienceDirect, Wiley, Sage, Scopus, Taylor & Francis, and Google Scholar. The publications in the form of reports, conference papers, working papers, discussion papers, journal articles, book sections, and textbooks were considered in this study. In total, 11 energy systems, including human and animal energy, solid biomass (firewood), hydropower, wind, geothermal, solar, nuclear, peat, coal, petroleum, and nonsolid biomass (methanol, hydrogen, ethanol, biodiesel, and biogas), are described. The current and future development and utilization of these energy systems have been described. The challenges for developing and utilizing these systems were elaborated on, and the solutions for their challenges were presented. Hydropower from the Nile River, being the main river for large hydropower plant construction, is the dominant energy system in Uganda. Nuclear energy will be the salvation for the country's electric energy supply in the near future. Therefore, Uganda needs to bet big on nuclear energy.

## 1. Introduction

Energy utilization is a prerequisite for both rural and urban communities' development. Modern energy access is a contributing factor to the nations' wealth growth [1]. Therefore, lack of energy access is normally termed energy poverty. Most sub-Saharan African countries lie under energy poverty with limited access to modern energy [2]. In Uganda, several policies have been put in place to alleviate energy poverty [3, 4]. For instance, there has been much analysis devoted to grid-connected distributed generation and rural electrification [5].

The global energy transition to 100% renewables by 2050 is ongoing, and Uganda is also in the race. This transition is not just science fiction but a demand for total leapfrogging into a sustainable future [6]. The main driver behind this is the need to combat climate change at all costs by reducing greenhouse gas (GHG) emissions to attain Sustainable Development Goals (SDG) [7–9]. The renewable energy development is a clean energy innovation approach that can enable climate-compatible growth in sub-Saharan African countries [10–12]. With the abundant and affordable resources in those countries, it is technically and economically practical that all power generation can be achieved using renewable energy resources by 2030 [13–16]. It is estimated that the contributory cost of technologies to ensure access to electricity for all Ugandans by 2030 ranges between $0.63 and $1.24 billion [17]. In general, government leaders have much power to shape energy transition. With good government policy, this transition can be attained, but with bad policy, this will end up to be stories for many generations to come. For example, populist leaders have long dispensed energy endowments for just political gains without accomplishing main goals [18].

Renewable energy is the biggest source of energy that can channelize energy systems in the direction of sustainability and supply security [19]. However, some studies pointed out that renewables have geopolitical and geoeconomics implications [20]. Besides, renewable energy generation faces a number of challenges, including climate dependence, technological, and distribution problems [21]. Nonetheless, renewable energy capacity in Africa is forecasted to reach 169.4 GW by 2040 from 48.5 GW in 2019, but this requires reevaluation (life cycle analysis) of the environmental impacts of renewable energy on the continent to inform mitigation decisions [22]. Hence, approaches to providing sustainable energy are not only the government leaders' strategy but also endless efforts from academic and policy circles [23].

The availability of dependable energy plays an essential position withinside the social, economic, and cultural transformation of society. In the case of Uganda, the energy area has suffered long-status facet constraints that led to suppressed demand and outages [24]. Long-term energy demand forecasting is crucial for any country, in particular for developing countries with rapid development of energy needs [25]. In this line, Uganda continues to politically and economically reform its energy sector, including a new legal and regulatory framework, based on which the previously vertically integrated monopoly, Uganda Electricity Board, was unbundled leading to public private partnerships. The Uganda government allows an enabling environment for private sector investments in generation and distribution of electricity, while transmission above 33 kV stays a public function via Uganda Electricity Transmission Company Ltd. (UETCL). The Electricity Regulatory Authority (ERA) was established to oversee the operations of all electricity operators, and the Rural Electrification Agency (REA) was launched to ensure that rural electrification is accelerated to accomplish preset electricity distribution targets in rural areas [26]. In the battle for increasing access to clean and renewable energy as well as accelerating electricity access to the unserved rural population in Uganda, the government of Uganda has licensed nine electricity distribution companies, including Kilembe Investments Limited, Hydromax, Pader-Abim Community Multi-Purpose Electric Cooperative Society, Uganda Electricity Distribution Company, Umeme Uganda Limited, West Nile Rural Electrification Company, Kyegegwa Rural Energy Cooperative Society, Bundibugyo Electricity Cooperative Society, and Kalangala Infrastructure Services Limited [27].

Currently, Uganda has a total of 24 power plants that produce and supply electricity to the national grid. These include 4 large hydropower plants and 11 small hydropower plants (1023.59 MW), 2 thermal or heavy fuel oil power plants (100 MW), 5 bagasse-based cogeneration power plants (63.9 MW), and 2 solar PV power plants (60 MW). To this end, there is no wind power share to the total national installed capacity. The distribution of electricity in Uganda has expanded enormously in over two decades, with legally grid-connected customers rising from 180,000 in 2001 to 1,643,288 in 2020, including off-grid clients. However, the country has an electricity import capacity of 20.5 MW from two power plants in the neighboring countries [28].

Uganda owns abundant energy resources, which are fairly distributed throughout the country [29]. These include hydro, biomass, solar, geothermal, peat, and fossil fuels with petroleum in an estimated amount of 6.5 billion barrels, of which 1.4 billion barrels are recoverable, which has been discovered in the western part of the country. However, currently, the country's electricity generation is mainly through hydropower. This alone is not sufficient to supply the whole population in the country, making electricity prices expensive besides other factors such as oil price fluctuation and the exchange rate [30]. Electricity in Uganda is not the most expensive but also not the cheapest compared to other sub-Saharan African countries [31, 32]. In 2018, Uganda's electricity price was below Rwanda's and Kenya's tariffs but above the tariff of Tanzania, South Africa, and Ethiopia [33]. Ang et al. [34] reported that highly volatile oil prices and unprecedented weather fluctuations have acted as significant shocks for electricity generation, influencing electricity pricing. Moreover, petroleum resources bring their own problem as it is very difficult for developing countries like Uganda to balance their economic development needs and contribution to combating climate change [35]. The rising electricity price in the country brings a great challenge to the consumers, especially the large industrial consumers. However, Ai et al. [36] urged that increasing electricity price has a good side in that it could force enterprises to carry out technological innovation and improve the efficiency of energy utilization. For instance, Uganda is planning to adopt an electricity prepayment billing system (EPBS) as an intervention to reduce nontechnical energy losses [37].

Rural electrification schemes, also known as distributed generation, often focus on generating power for electric lighting and, more recently, phone charging [38]. In most sub-Saharan African countries, the rural electrification is strengthened through the formation of rural electrification development initiatives [39]. Moreover, a number of organisations or initiatives, such as GIZ and GETFiT, play a great role in promoting energy access to the local community [40]. In this regard, regional and international cooperation can standardize electricity access benchmarks and facilitate technology transfer through existing or more improved instruments [41]. For example, a number of project developers are present in sub-Saharan African countries with a focus on the mitigation of carbon footprint. These project developers are classified into neodevelopmental (in Tanzania) or liberal neodevelopmental (in Uganda) [42].

The government intervention is of paramount importance for reducing electricity and petroleum prices. Particularly in rural areas, through the rural electrification program, the price of electricity is reduced, and the suppliers are compensated. This is done through a subsidy policy [43]. Moreover, incorporating the heterogeneous nature of price elasticities into pricing policy can help decrease electricity demand-supply mismatch and inequality in electricity consumption [44]. In most developing countries, electricity pricing is determined by the mechanism called Automatic Tariff Adjustment [45, 46]. On the other hand, petroleum prices are very flexible since Uganda does not refine its oil though there is a large amount of crude oil deposit. It has to export the crude oil and import the refined ones, and thus the price structure will depend on many factors, including transportation, pandemic, and inflation [47–49]. For instance, the COVID-19 pandemic led to an oil price shock, which hit oil-exporting developing countries as well as the importing countries [50]. Raghoo and Surroop [51] cited that transportation and taxation policies are the major parameters for determining petroleum prices.

With electricity being the main form of energy today, it has increased the complexity of life today. In Uganda, electricity generation is mainly through hydropower which puts the country in the bottleneck of overdependence on one source of energy [52]. However, there are many energy systems out there (such as solar and biomass) that the country can use to diversify its electricity generation. Therefore, the need to assess the level of development and utilization of various energy systems has been the underlying question for this present study which has not been addressed by the previous studies (Table 1). The contributions of this paper are threefold: (i) describing the current status, (ii) discussing the challenges and mitigation, and (iii) providing the future trends of energy systems development and utilization in Uganda. The present paper is structured as follows: Sections 2–13 present the review of the different energy systems.  Section 14 provides a brief discussion on each energy system. Section 15 concludes the paper.

## 2. Methodology

In this study, a critical and comprehensive literature search was conducted to uncover the current status of energy systems utilization and development in Uganda following the procedures used by previous studies [64–66]. The published literature in the forms of book sections, conference papers, peer-reviewed journal articles, theses, dissertations, reports, white papers, discussion papers, and working papers. The more general Google search engine was used to obtain scholarly, nonscholarly, and business published literature. Electronic databases such as Google Scholar, Science Direct, Wiley, Taylor & Francis, and Springers were used to narrow the search for scholarly articles and book sections or chapters. The search terms employed include “Hydropower and Uganda,” “Human energy and Uganda,” “Animal energy and Uganda,” “Wind energy and Uganda,” “Solar energy and Uganda,” “Peat energy and Uganda,” “Nuclear energy and Uganda,” “Geothermal energy and Uganda,” “Nonsolid biomass energy and Uganda or biogas and Uganda or co-generation and Uganda,” “Solid biomass energy and Uganda or wood fuel and Uganda,” “Petroleum energy and Uganda,” “Coal energy and Uganda,” and “ethanol and Uganda.”

## 3. Human and Animal Energy

### 3.1. Current State of Utilization

**Figure 1:**
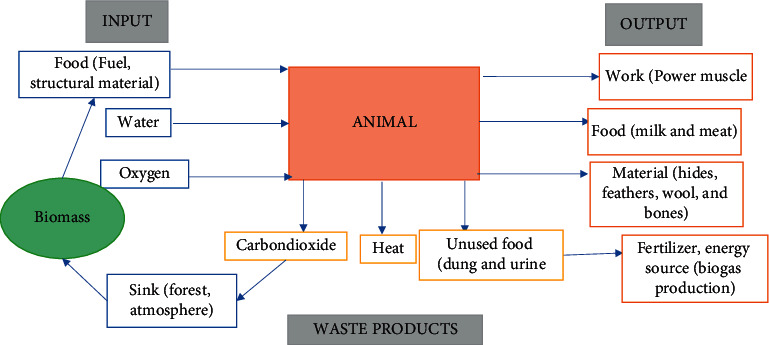
Input-output block for working animals, adapted from Fuller and Aye [67].

In Uganda, human and animal power still contribute a significant proportion of the energy used in the rural areas. The technologies include merry-go-round generators in schools, hand crank lighting in hospitals and health clinics during electricity outages, and bicycle generators for off-grid businesses. They are the most important energy sources for the population and are the largest single contributor to renewable energy sources [67].  Figure 1 clearly demonstrates the concept of harnessing animal power as an input-output block diagram.

The human and other animals' energy is largely employed in agriculture, crop processing, transportation, construction and fabrication work, and industrial manufacturing process (operating machines). Agriculture still dominates the proportion of human and animal power, followed by transportation (Table 2) [68], whereby human and animal power is the elementary level of mechanizing agricultural and forestry operations. These agricultural operations include, but not limited to, the following: clearing of vegetation/stubble, including cutting trees (machetes, axes, hand, and chain saws), land preparation and soil manipulation (blade and tined hoes and pickaxes), seeding or planting (broadcasting by hand or seed fiddle, dibbling, jab planting, and use of single or multirow seeders), weeding (weeding hoes and cultivator weeders), fertilizer and manure application, crop protection (manual or power knapsack sprayers and dusters), harvesting (scythes, sickles, knives, drags, forks, and rakes), processing and preservation of food and feed (baling, mechanical threshing, manual and mechanical shelling, and grinding), transportation (head or shoulder load, backpack, handcarts, and bicycles), and other secondary tasks such as operation of hand and treadle pumps to lift water. Generally, human energy is a forgotten power option and, if implemented in countries with average electrical power consumed below 20 W/capita, would have a direct impact on human development [69].

### 3.2. Challenges and Their Possible Mitigation

Despite the fact that human and animal power is the most common energy used by the people in Uganda, they have some drawbacks. The cost of the improved technologies is a significant barrier to human and animal power adoption. For instance, many people in the villages are carrying loads on their heads or backs, which means that rural people are unable to afford simple technologies such as wheelbarrows, handcarts, bicycles, and animal-drawn carts. Secondly, there is high isolation of those who would benefit most from the introduction of more efficient hand, and animal-powered technologies are also a major factor influencing the steps in a successful technology transfer process [68].

The following can be possible mitigation for human and animal power drawbacks [71]: (i) proper tools to harness the power of humans and animals, (ii) appropriate and sufficient feed to ensure health and growth, as well as energy for work, (iii) adequate healthcare and prompt provision of veterinary services in case of injuries and sickness to aid in resistance to disease, (iv) appropriate well-fitting equipment for working, prevention of injury while working, and so on, (v) prevention of overstraining, allowing human and animals to rest from work while sick or injured, and avoiding putting animals to work in adverse ambient conditions, (vi) ensuring observance of laws to prevent the misuse and abuse of draught animals, (vii) use of modern equipment and methods for surgical treatments and slaughter, (viii) allowing animals the freedom to satisfy natural instincts, adoption of human methods for nose-roping, shoeing, branding, and dehorning, and so on, and (ix) development of publicity and education programmes by animal welfare organisations.

### 3.3. Future Trends

There is a dire need to increase the energy supply of those who rely on traditional energy sources in order to improve their quality of life and reduce the drudgery and hardship of everyday life. Human and animal energy is the forgotten renewable source of energy that is environmentally friendly and sustainable energy supply system. However, the technologies used to harness this system are simple and reliable but need much attention [72]. There is a need to foster agricultural productivity by heightening the electricity access to rural populations through rural electrification development [73].

## 4. Solid Biomass Energy

### 4.1. Current State of Utilization

**Figure 2:**
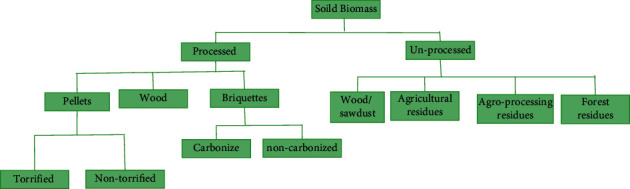
Classification of solid biomass energy.

In Uganda, the use of low-grade forms of energy (especially traditional or solid biomass fuels) accounts for more than 90% of total energy consumption. With a large number of refugees in the country, energy supply is so limited, especially energy for cooking (firewood) [29]. Solid biomass can be classified into processed and nonprocessed biomass as depicted in Figure 2. The use of nonprocessed biomass sources of energy or solid biomass (i.e., crop residue and wood fuel) illustrates energy poverty among households [74]. In addition, wood fuel is used extensively for process heat and to fuel brick-burning, tea drying, cement, titles, and lime production. The major sources are hardwood plantations, which consist of eucalyptus (50%), pine trees (33%), and cypresses (17%). The current available sustainable wood biomass supply is about 26 million tons. The annual theoretical potential production of agriculture residue ranges from 1.186 million to 1.203 million tons. The only subsector that utilizes biomass residues for electricity production yet is the sugar industry [29, 30].

It is estimated that 3 billion people, most of whom live in Asia, Africa, and the Americas, rely on solid fuels (i.e., wood, crop wastes, dung, and charcoal) and kerosene for their cooking needs. Over 700 million people in sub-Saharan Africa depend entirely on solid biomass fuels, especially unprocessed ones, and use simple or traditional cookstoves in poorly ventilated kitchens, which results in high indoor concentrations of household air pollutants such as fine particulate matter, carbon monoxide, and Polycyclic Aromatic Hydrocarbons (PAHs)[75–78]. Exposure to household air pollutants from burning this unprocessed biomass is associated with several health problems as well as premature [79, 80].

Processing solid biomass into charcoal has been one of the strategies to provide less emission cooking fuel for urban people. However, charcoal production from the forest is often not effectively regulated and thus contributes to forest degradation, deforestation, and GHG emissions, as well as climate change [81]. Moreover, charcoal also has adverse health effects on household users [82]. Despite the efforts to minimize deforestation, forests will continue to be a critical source of domestic energy for households in developing countries [83, 84]. Therefore, there is a need for a cheap and efficient fuel alternative that can be easily adopted by the local population such as carbonized briquettes from agricultural residues and torrefied pellets from MSW [85, 86]. Besides, a transformative new approach to facilitate access to affordable, reliable, sustainable, and modern energy for cooking by leveraging rapid progress in rural electrification or distributed generation and falling prices of solar PV and lithium-ion batteries for battery-supported electric cooking is inevitable [87]. Furthermore, strategies for reforestation, dissemination of improved cookstoves, relieving supply side constraints for modern fuels, and staggered payment options to lower the cost of entry for modern fuels are better solutions to overdependence on the solid biomass for households' cooking [88, 89].

### 4.2. Challenges and Their Possible Mitigation

Firewood or wood fuel is the main source of heating and cooking in rural and urban areas. The high demand for fuel wood has resulted in the depletion of forests and exacerbates land degradation. This type of energy source is associated with higher levels of indoor pollution, time allocations especially by women and children for its collection, unreliability of supply, and local environment degradation. The use of firewood is therefore a detrimental factor in welfare improvement and constraint to the achievement of all the eight Millennium Development Goals [90].

The above challenges can be mitigated by the use of an alternative energy source or processed wood fuels such as charcoal, briquettes, and other agricultural residues, which are very important for reducing overreliance on firewood [91]. The charcoal is typically produced in low-efficiency Earth kilns in rural areas, and high losses are experienced throughout the value chain. LPG, natural gas, and electricity are used mostly by the high-income groups, normally in urban areas [92]. However, massive use of these energy sources can end energy poverty in the country [93]. With these regards, it can be noted that energy use varies considerably depending on income and geographical location of the household. Promotion of waste as a business by the government to accelerate the processing of biomass wastes (value addition) for household cooking [94].

### 4.3. Future Trends

The efficiency of the traditional and institutional cook stoves is being improved to reduce the consumption of firewood. Moreover, the efficiency rates in terms of energy consumption industries such as tea, tobacco, lime, and brick-making are being improved. Importantly, the key tool for this is the energy audits to identify potential measures to improve energy efficiency. Generally, a sustainable national grid and small-scale solutions like efficient biomass stoves, biochar, gasifiers, and biogas installations are highly recommended for future development as a lucrative approach for securing sustainable and clean energy in Uganda [95].

## 5. Hydropower Energy

### 5.1. Current State of Utilization

There are three kinds of hydropower generation plants: (i) run-of-river, where the power is generated by the flow of a river, (ii) reservoir, where the power is generated by the release of stored water, and (iii) pumped storage, where stored water is backed up into the reservoir in order to be pumped again [96, 97]. Small-scale hydropower stations are typically of the run-of-river type, while the large hydropower plants are of the reservoir type [98]. The large hydropower potential in Uganda along River Nile is estimated at about 2000 MW. With only 380 MW developed at Nalubaale and Kiira and 250 MW under development at Bujagali, the unexploited potential is well over 1300 MW.  Table 3 shows the seven (7) operational large hydropower plants along River Nile, River Mahoma, and River Achwa, with a total capacity of 926 MW [99]. In Uganda, most of the hydropower projects are mainly commissioned by the private investors, for example, independent power projects and emerging Chinese-funded projects [100].

Small hydropower (<20 MW) projects are mainly not on the Nile River and have not been fully exploited. These are important sources of electricity for areas not covered by the national grid. Even though the cost per unit of electricity from isolated small hydropower plants may be higher than that from the national grid, they could sustainably contribute to poverty reduction in households in isolated areas.  Table 4 shows the small hydropower sites available for development in Uganda. There are currently 25 small hydropower plants that are operational with a total capacity of 195.5 MW.

### 5.2. Challenges and Their Possible Mitigation

In this section, the challenges and mitigations for hydropower development in Uganda have been described. Hydropower projects require huge initial investment costs because of civil engineering work cost, equipment cost, land compensations costs, and transmission system cost [102, 103]. In order to minimize these challenges, the engineering hydropower policy in Uganda should consider large dam projects with a smaller reserve surface area in comparison with power generated [104, 105].

Further, the low human and institutional capacities to manage design, construction, and management of hydropower plants are another barrier to hydropower plant development in Uganda. This can be reduced by developing local capacity in order to minimize dependency on costly foreign expatriates; conditions should be set in the agreements with hydropower developers to train local manpower to manage the hydropower plants, and clear deadlines should be established when the local manpower should take over from the foreign expatriates. Furthermore, specialized curriculums in collaboration with industrial partners should be developed by Uganda's Universities in energy technologies with a focus on hydropower and other energy resources in the country [106, 107].

In addition, there is high resistance to hydropower projects from the community because hydropower development process is considered a threat to livelihoods, ecosystem, and biodiversity as it brings about human displacement and natural resources degradation [108]. To solve the problem, the government of Uganda needs to establish a clear and well-defined resettlement plan for natives that are to be displaced by the establishment of hydropower generation plants. The resettlement plan should be discussed by the affected populace and the project development partners to come up with a win-win resolution [109, 110].

Lastly, hydropower generation depends on the run-of-river water, which has a direct relationship with the amount of water entering and leaving the rivers. Climate change has a major impact on the electricity infrastructure in the country due to weather extremes like floods that damage hydropower spillways and damage the electricity transmission infrastructure [111, 112]. Additionally, reduced inflow flow due to climate change has led to the failure of many hydropower projects [113, 114]. Ugandan rivers are being tapped into reservoirs as a resource for the generation of hydropower through the construction of dams, which becomes competitive with other uses such as irrigation, freshwater for households, and fishing [115]. This can be minimized by considering a massive investment in small- and medium-scale hydropower instead of large-scale hydropower generating plants. This is because the small hydropower plants depend on lower water levels to run turbines and hence are less affected by reduced water levels. Therefore, it is very important to forecast the water level using software and other advanced forecasting tools [116, 117].

### 5.3. Future Trends

In order for the country to meet its energy demand, many hydropower plants are under construction, and some are being proposed. So far so good. Five (5) large hydropower plants are under construction, while three (3) have been proposed with a combined capacity of 2514 MW as shown in Table 5. Five small hydropower plants (32 MW) are under construction, and twenty-seven (125.6 MW) have been proposed as presented in Table 6 and Table 7, respectively, while thirteen (5.66 MW) are under preliminary studies, and some of them have no studies as depicted in Table 8.

## 6. Wind Energy

### 6.1. Current State of Utilization

Wind energy is not yet developed and is presently an unused resource in the country for electricity generation. However, the wind speeds thought to be commercially viable are found in Tororo, Pader, and Nakapiripirit Districts with average speeds ranging from 7 to 9 m/s at the height of 80 m. Nevertheless, the wind speed in most areas of Uganda is moderate, with average wind speeds in low heights (>10 m) between 2 m/s to 4 m/s. Therefore, the wind energy resource in Uganda is only sufficient for small-scale electricity generation and for special applications, such as water pumping, mainly in the Karamoja region [118]. The other current use of wind energy has been identified to be used for small-scale irrigation [120].

### 6.2. Challenges and Their Possible Mitigation

The following are the challenges hindering the wind energy development in Uganda and their mitigation approaches. There are currently insufficient wind resource data within the country, which is demanded by wind energy projects. These data should be consistent and reliable data from different locations within the country. In order to overcome this, there is a need to develop a wind energy data centre to collect and analyze wind data parameters across the country [27, 121]. Secondly, there are challenges regarding wind variability and intermittency, and this is a common natural occurrence in any geographical location across the world. This challenge affects power generated, may cause turbine faults, and can compound inaccuracies in load forecasting.

Development of wind power farms requires a skilled workforce for wind resource assessment, infrastructure installation, operation, and maintenance, especially in the implementation of large wind projects, which is currently lacking in the country. This can be minimized through capacity building, which can be achieved by deliberately advancing long-term capacity and technical know-how in wind power technologies through training, research, and development [121].

The technologies used for wind power generation and supply are very expensive. An initial cost of investing in wind power is approximately 80% of the total project costs. Additional costs are operation, maintenance, and insurance. The high investment cost can be minimized by a financial risk transfer approach. This constitutes instruments that transfer a proportion of the risk to public sector agencies and include Feed-in Tariffs (FiTs), subsidies, Feed-in Premiums (FiPs), auctions, green bonds, equity financing, and/or hard loans [27].

The volatility of wind energy interferes with the system's capability to control electricity supply. The control of wind energy intermittency due to climate change is even more complex with weak grid infrastructure due to inexistent or sufficient high-power voltage transmission. Wind energy generation is categorized as nonsynchronous and is associated with instability effects on an electricity system due to low inertia levels. This weak infrastructure can be reduced by expanding grid infrastructure that could ease connectivity to the grid by independent wind energy producers, attract private investors, and broadly increase efficiency in the generation and distribution of electricity [122].

### 6.3. Future Trends

The wind energy development in Uganda has remained so low, and it is still being harnessed in a traditional manner such as windmill, winnowing, and many others. There is an effort to develop and install wind turbines in some of the selected sites across the country. This is aimed at achieving renewable energy to back up the hydropower energy, which is currently the dominant clean energy in the country.

## 7. Solar Energy

### 7.1. Current State of Utilization

In the current situation, only 28% of the population have access to electricity which is not even reliable in the country. However, Uganda presents a huge market potential for alternative technologies to provide electricity, such as solar energy and photovoltaic (PV) systems [123]. Solar energy is underutilized in the country, although this is slowly changing. Overall, the projected solar penetration in a different part of the country by the year 2021 was 6.1%, with the total annual energy estimated at 69.52 GWh [124]. The use of solar PV started in the 1980s and has been utilized for lighting, vaccine refrigeration in health centres, communications, and signaling for the railways and for telecommunication. In 2014, two 10 MW solar power stations (Tororo Solar Power Station and Soroti Solar Power Station) in the east of Uganda were licensed by the ERA [125]. There exist several PV solar panels with different market segments, including Pico and microsolar home system, solar home system, standalone institutional solar PV system, solar PV minigrids, and telecommunication and lighting PV solar systems that are being installed [126]. Pico and microsolar systems are mainly adopted by the locals in the villages where there is no national grid for electricity [127]. The other minigrid PV solar systems include Xsabo solar plant (20 MW) and Mayuge solar PV plant (10 MW). Among the different solar PV systems, rooftop solar PV systems emerged as the best option, followed by ground-mounted solar PV systems [128]. In the rural population, the electricity generated by the solar home PV systems is mainly used in household application such as cellphones, lighting, and radios [129]. Solar energy has been an attractive area of study among researchers in the recent past.  Table 9 shows examples of studies on solar energy in Uganda.

### 7.2. Challenges and Their Possible Mitigation

The main challenge with solar energy is the environmental impact. Solar energy does not pollute air and water or cause greenhouse gases. It can have a positive, indirect effect on the environment. Using solar energy replaces or reduces the use of other energy sources that have larger negative effects on the environment. However, some toxic materials and chemicals are used to make the photovoltaic (PV) cells that convert sunlight into electricity. Some solar thermal systems use potentially hazardous fluids to transfer heat. Leaks of these materials can harm the environment and cause health effects to human beings and animals. However, environmental effects from solar energy technologies are usually minor and can be minimized by appropriate mitigation measures. The potential environmental burdens of solar energy are regularly site-specific, depending on the size and nature of the project. This can be minimized by proper site selection. The selected sites should not interfere with land farming and other land uses. Some of the mitigation strategies concerning community resistance and investments cost can be borrowed from the previous energy system such as hydropower and wind energy [27, 105].

### 7.3. Future Trends

With the need to achieve 100% renewable by 2050, Uganda will have to develop its solar energy four times the existing capacity [92]. This can be simply put as the need to use solar and other renewable sources of energy will be no more an alternative but a must-do thing in the near future. The government of Uganda has therefore started a partnership with the private sector energy providers that can build solar plants in Uganda and learn from them for the agreed duration of time. Solar hybrid system can be abetter energy techonology for Uganda in the near future [137, 138]. Therefore, the economic viability of solar hybrid systems needs to be investigated in the case of Uganda, for instance, floating solar PV and hydropower hybrid system, biogas-solar PV system, PV-storage-diesel generators, and many others [139]. Solar energy for thermal application plays great for postharvest management as it is used for drying [140]. Hence, the development and utilization of solar dryers should be expanded in rural Uganda. The use of software for hybrid system optimization [141] and long-term energy planning optimization model with integrated on-grid and off-grid electrification should be extensively researched [142].

## 8. Geothermal Energy

### 8.1. Current State of Utilization

The global geothermal energy market and utilization are constantly increasing with the US remaining the largest national market [143–146]. The potential and status of geothermal energy development vary from country to country and region to region [147]. Uganda still has no geothermal energy in operation. Ever since the quest for geothermal potential in Uganda began, more than 40 geothermal sites have been studied for their prospect's parameters like temperature, chemistry of reservoir, natural heat transfer, and fluid characteristics to identify specific project areas and prioritize those for more detailed investigation. So far so good, three major potential areas for geothermal energy have been discovered as detailed in Table 10 [118], while the latest site discovered is Panyimur geothermal, which is located in Pakwach District.

### 8.2. Challenges and Their Possible Mitigation

There are 10 challenges that hindered the geothermal development in Uganda: (1) land access barriers and competition, (2) diversification of Uganda's energy mix, (3) large investment costs, (4) lack of awareness and information, (5) government policy, incentives and institutional challenges, (6) inadequate research and development, (7) inadequate human capacity and training, inadequate infrastructure to support geothermal energy development, (8) inadequate infrastructure to support geothermal energy development, (9) shortage of financial resources, and (10) sociocultural and environmental challenges [148, 149].

Several mitigation approaches have been proposed; however, most of the solutions to the challenges are similar to the other energy systems described earlier. Some of the countermeasures for the abovementioned challenges include financial and subsidy incentive to individuals and communities as well as private organisations for the development of the geothermal energy project and community participation/ownership of geothermal energy projects for security and infrastructure. Loan facilities can be sought from African Development Bank as well as the global environmental facility. Regular environmental audits and environmental systems strengthening and streaming to ensure proper use and restoration of existing ecosystem services [148].

### 8.3. Future Trends

Until now, all the studied prospects have not yet reached an exploration stage suitable for the targeting and drilling of deep exploration wells. Expected temperatures are suitable for electric power generation using ORC plants, subject to the confirmation of the existence of reservoirs suitable for industrial exploitation. Geothermal energy is one of the possible alternative renewable energy sources in Uganda, which could supplement other sources of energy. Therefore, the country is not giving up on it. The development of technology and skills to extract the geothermal energy from the sites discovered is in progress.

## 9. Nuclear Energy

### 9.1. Current State of Utilization

Nuclear energy is considered a valuable option for the decarbonization of power generation, as it is produced from noncarbon resources [150]. Therefore, it plays a major role in meeting the future global energy needs and mitigating the threat of climate change [151]. Uganda is among the African countries with a high potential for nuclear energy development because of the availability of uranium reserves [152–155]. However, Uganda's energy scenario is quite different from other African countries such as Ghana, Libya, and Egypt. It has a nuclear power potential of 24000 MW, but preliminary findings indicate that 50000 km^2^ of estimated uranium can be found around Buyende, Nakasongola, Mubende, Kiruhura, Buhweju plateau, and Lamwo. There is a need for proper investment planning by the government as the unit cost of developing 1 MW of nuclear energy is $6 million. However, the government efforts are to build a 1000 MW power plant in the medium term and 2000 MW in the long run. In total, the government is planning to invest in energy infrastructure and raise generation capacity to 3500 MW in the near future, and it also seeks to increase per capita consumption from the current 215 kWh to 674 kWh over the medium term [156]. This will be unprecedented energy reform in the country due to its abundance.

### 9.2. Challenges and Their Possible Mitigation

There are numerous challenges that are debarring atomic energy development in Uganda besides the danger and the fear of nuclear energy. These challenges include but not limited to the following as highlighted [157]: (i) There is a very high initial capital cost of building a 1000 MW nuclear power station in Uganda, averaging almost $6 billion (in 2020 dollars). (ii) There is insufficient public awareness of nuclear power development. Worse still, technical information is inadequate, and data is insufficient to accurately assess the availability and true potential of nuclear energy. (iii) There is strong competition with other energy sources. (iv) Uganda which has no policy on nuclear energy is not expected to have a waste management policy on nuclear wastes, which is quite dangerous for the country. (v) Nuclear technology is very complex and demanding that requires specialized knowledge and excellence in human performance, which has never been developed in the country. (vi) Another critical barrier to the development of nuclear energy development in Uganda is the absence of the enabling infrastructure (grid unreliability) in the form of transmission and distribution lines that can transmit electricity to remote places, and because of this limitation, they resort to rudimentary technologies used in most of the rural places in Uganda which are essentially small and very inefficient. (vii) Presently, there is limited research effort by the government of Uganda in nuclear energy. Notably, there is no nuclear energy research and development program that is reinforced with government funding. (viii) One of the most critical challenges is that politics and geopolitical risks are embedded in nuclear energy development [157–159]. (xi) The fear of environmental concern related to nuclear energy production and uranium mining is among the challenges contributing to the delay of nuclear development in the country [160]. Further challenges of nuclear power development are well-elaborated by the previous studies: Adams and Odonkor [161] and Ansah et al. [162].

Several countermeasures to the challenges have been pointed out. These include the following: the government of Uganda may undertake an energy subsidy reform by transferring subsidies from fossil fuels to nuclear energy technologies. In addition, there is a need to regularize manufacturing processes in order to promote nuclear energy technology in Uganda. Most of the solutions, such as capacity building and feed-in-tariffs policies, are similar to other energy systems [163].

### 9.3. Future Trends

In the face of growing energy needs arising from the rapidly growing population, there is a need to find alternative clean, efficient, reliable, and affordable sources of energy in Uganda which can meet this need. As such, nuclear energy has been considered a good fit that could cover this unprecedented energy demand as well as soothing socioeconomic activities in the country [156].

## 10. Peat Energy

### 10.1. Current State of Utilization

Peat is the surface organic layer of soil, consisting of partially decomposed organic material, derived mostly from plants, which has accumulated under conditions of waterlogging, oxygen deficiency, acidity, and nutrient deficiency. Peatlands are areas of landscape, with or without vegetation, that have a naturally accumulated peat layer at the surface [164]. The peatlands area in Uganda is projected to be about 4000 km^2^, and the average thickness of peat deposits is estimated to be about 1.5 m, with the total peat volume to be 6000 million cubic metres, while the average dry bulk density is estimated to be around 100 kg/m^3^ and a net calorific value of 17 Giga Joules/tonnes while theoretically, peat volume corresponds to about 250 million tonnes of oil equivalent (Mtoe) [105]. Interestingly, peat energy can be a promising source of electricity for Uganda because peat-to-power technology is cost competitive compared to the electricity utility price [165].

### 10.2. Challenges and Their Possible Mitigation

The damaged peatlands in the country are already releasing almost 3.7 megatons of CO_2_ equivalent each year, which is very dangerous to the environment. Moreover, these emissions are likely to increase with further peatland deterioration as a result of climate change [166]. Generally, peatlands need more studies and long-term monitoring in relation to vegetation changes and corresponding ecosystem services such as GHG, water quality, and flooding. This is helpful in supporting further financial investment. Sharing good practice on peatland management and scientific information across peatland countries is an important objective [166].

### 10.3. Future Trends

Kabale Energy Limited is the first company entrusted by the ERA to undertake studies necessary for generation of approximately 33 MW using peat resource in Kabale District. Meanwhile, ERA is currently processing a permit extension application for the said project. There is hope that the total available peat resource volume will be adequate for generation of about 800 MW for the next 50 years. However, the available peat resources are dispersed mainly to Western and South-Western Uganda, where the desired characteristics are better than in other regions [118].

## 11. Coal Energy

### 11.1. Current State of Utilization

There is currently limited study on the existence, consumption, and production of coal in Uganda. This implies that there is no coal deposit and utilization in the country [167].

### 11.2. Challenges and Their Possible Mitigation

Coal production and uses in Uganda have not yet commenced; however, some challenges are attributed to the use of coal as derived from the coal dominant countries such as China and the USA [168].

Price inflation is a problem associated with coal in that depletion of the highest-quality, easiest-to-mine coal leads to higher prices. The price of delivered coal is also sensitive to oil price increases because diesel fuel is an important input for mining and transportation. Price inflation can be solved by the value diversity in fuels, technologies, and suppliers in integrated resource planning; this will reduce the tendency of overdependency on coal only by investing in solar, wind, and geothermal, among others [169].

Environmental constraints and costs are associated with meeting new pollution-control requirements. Environment pollutants such as GHG emission, particulate matter, and NOx due to coal mining and combustion has become a global concern since the world is eyeing carbon-free production and operation; therefore, new and pending environmental rules are expected to increase substantially the costs of operating existing or building and operating new coal plants, and some of the technologies are proposed to better manage emissions [170, 171]. Fully evaluate pollution-control investments for existing power plants and secure option values by evaluating practical options, investigating those that are most promising, and procuring those that produce the most value under the broadest range of plausible future conditions. This will prevent issues associated with environment pollution and global warming due to carbon emissions.

There is a health issue concerning the use and production of coal. Coal kills people and causes disease from coal-fired power plants causing 23,600 premature deaths, 21,850 hospital admissions, 554,000 asthma attacks, and 38,200 heart attacks every year. Additionally, coal kills jobs compared to other renewable energy sectors like wind and solar. In America, the wind sector employs more workers than the coal industry. Investing in wind and solar power would create 2.8 times as many jobs as the same investment in coal; mass transit and conservation would create 3.8 times as many jobs as coal [172]. Policy drivers like the introduction of stringent environmental and safety regulations are less favorable against coal but can help to reduce safety violation and hence decreases exposure to coal [173]. The more radical approach to mitigate the negative and harmful effects of coal usage is through cancelling new coal power plants [174].

### 11.3. Future Trends

Since there is no much information related to coal deposits in the country, the future trend will only rely on further exploration with technology that will be available in the future. With constant exploration, maybe one-day coal deposit can be found in the country.

## 12. Petroleum Energy

### 12.1. Current State of Utilization

Being a landlocked country, about 85% of Uganda's petroleum imports are routed through Kenya and 15% through Tanzania [32]. However, commercially viable deposits of oil around Lake Albert in western Uganda were first discovered in 2006 [175]. Oil exploration and development in Uganda were triggered by the high international oil prices between 2004 and 2014 [176]. The late discovery of oil made Uganda becomes one of the region's newest petrostates [177]. Therefore, this led to the creation of new legal frameworks for oil and gas in Uganda in 2013 to put in place local content policies [178]. The presence of oil in the country will gradually change the petroleum industry in the near future. The oil and gas development in Uganda is being championed as a key to a “better life” [179]. Nevertheless, the full exploitation of the deposits might require the construction of an export pipeline to the Indian Ocean coast at Tanzania or Kenya coastal areas, although other possibilities are being examined [180].

**Figure 3:**
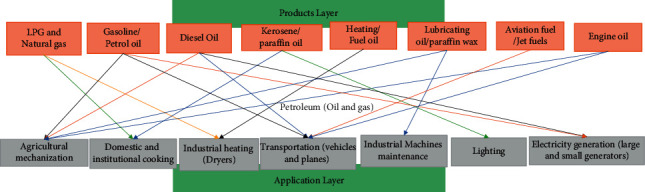
Applications of petroleum products.

The transport sector is the major consumer of fossil fuels and accounts for about 75% of the fossil fuel import bill [32], while Liquefied Petroleum Gas (LPG) (0.06%) and electricity (0.45%) make up a relatively small portion of overall household energy demand. Overall, the combined diesel and LPG contribute 3.2% of the sector's energy consumption in the country. In general, households that use LPG consumed approximately 31% less charcoal than those not using LPG [181]. This illustrates the benefit of petroleum product utilization in minimizing overdependence on solid biomass [182]. Petroleum products have been used in several areas of application as illustrated in Figure 3.

### 12.2. Challenges and Their Possible Mitigation

**Figure 4:**
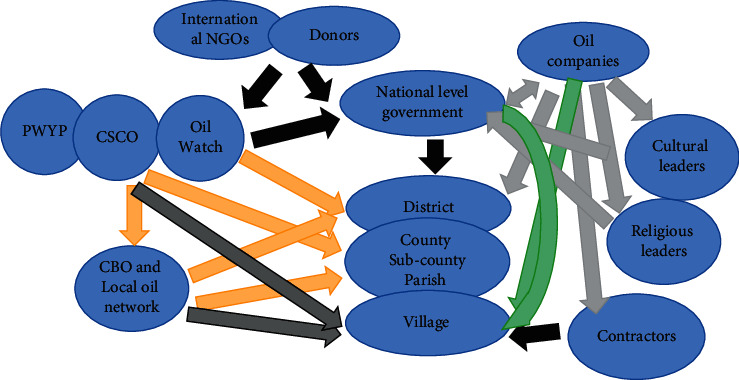
Space for governance in Uganda's oil sector. CSOs: coherence among civil society organisations; CSCO: civil society coalition on oil and gas; PWYP: publish what you pay; CBOs: community-based organisations [183].

Several challenges are hindering the development and utilization of petroleum energy. These include but not limited to the following: weak local government capacity for oil resource governance (Figure 4) and lack of industry-driven interaction with the local people [183]. The cost of investment in petroleum is too high, considering right from exploration and extraction to transportation, leaving alone refinery, which Uganda cannot handle with the current technology. For instance, the construction of Uganda section of the East African Crude Oil Pipeline (EACOP) will cost over USD $3.5 billion in total to transport crude oil from Hoima in the Albertine Graben region of Uganda to Tanga in Tanzania, a distance of 1443 km [184]. Besides, the construction of an oil pipeline can result in so many environmental and social impacts, including physical displacement, resettlement, economic displacement, disputed valuations, delayed compensation, livelihood disruption, food insecurity, uncertainty, fear, and anxiety [184].

The fear of environmental degradation (especially noise pollution, soil erosion, and wildlife disturbance) caused by oil and gas exploration activities is among the challenges for petroleum development [185]. Moreover, oil extraction is associated displacement of people living around the area of vicinity [186]. Next, the fear of petroleum pipeline fire and explosions, which can be catastrophic and lead to dangerous destruction of properties and loss of human and animal life, is also among the challenges [59].

Oil and gas development is associated with geopolitics [187]. Therefore, present oil in some countries is just a resource curse with endless instability [188]. In this regard, political leaders have a great influence on the success of petroleum development and utilization in the country. So, political leaders who are known to be bureaucrats can spend natural resource revenue in ways that entrench their political power but undermine longer-term economic development [188], hence, leaving their countries in constant and miserable energy poverty level. Therefore, greater stakeholder involvement can be helpful in alleviating the fears of shaping negative expectations and create conditions necessary to eradicate bureaucracy among the political leaders hence avoiding the energy resource curse [184, 189]. In addition, political and institutional innovation is inevitable to address the challenges related to oil development in the country [190]. Above all, some of the mitigation strategies of the challenges are similar to ones described in the case of the coal energy system.

### 12.3. Future Trends

Uganda started its oil extraction at Albertine, and the creation of a pipeline to Tanzania was proposed by the government. The government also plans to develop its own processing plant. Currently, the country is exporting unprocessed crude oil. Therefore, the future development will be to establish an oil refinery plant in the country. Recycling petroleum products such as polythene, PET bottles, and polypropylene into diesel can also be beneficial to the environment as well as increasing fuel supply in the country. This is attracting a number of researchers in the present days on how diesel fuel oil can be obtained from municipal plastic wastes, and it is efficient to use in transportation [191]. It can be concluded that the Uganda oil project is generally profitable and that deferring oil production is justified except in the cases where the net convenience yield or cost inflation is high [192]. The people mindset toward becoming a Petro-citizenship needs to be assessed because it might also affect petroleum development in the country [193]. Nevertheless, continuous discovery of oil deposits and estimation of available oil reserves is of great interest to the country [194].

## 13. Nonsolid Biomass Energy

### 13.1. Current State of Utilization

**Figure 5:**
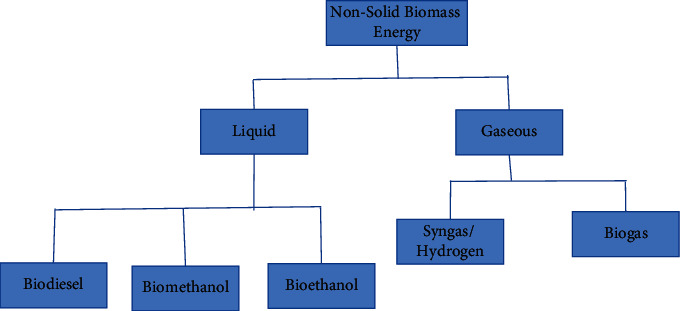
The classification of nonsolid biomass energy.

The nonsolid biomass energy includes liquid and gaseous biofuel such as biogas, bioethanol, biomethanol, biodiesel, and hydrogen as depicted in Figure 5. The transition from traditional biomass (wood and charcoal fuel) to modern biomass and biofuel production and consumption is a main focal area of the government to go green with sustainable energy systems by 2050 [195]. There are modern biomass energy systems called cogeneration which involves using biomass for general electricity as alternative energy [196]. So far, Kakira Sugar Works Limited and Kinyara Sugar Limited are generating electricity for sale to the national grid from bagasse, providing 12 MW and 5 MW, respectively, as of 2010. Generally, biomass cogeneration from agricultural wastes is seen to hold particular promise as a technology for the country. Biogas is among the most globally adopted biochemical conversion technologies for waste-to-energy technology [197, 198]. However, biogas digester distribution is still very limited in Uganda. The biogas implementation process started way back in the 1990s, and 50 digesters were installed in five districts (Iganga, Kabarole, Mbale, Mpigi, and Tororo) in the country by 2004 [105, 167]. There is also already power being generated from wood gasifiers at Muzizi Tea Estate (250 kW) and Mukono gasification system (10 kW) [199].

Besides biogas, ethanol production is also developing at a rapid pace. So far, there are three companies producing ethanol from several feedstocks. For instance, Kakira Sugar Works Limited in Jinja and the Sugar Corporation of Uganda Limited in Lugazi already have installed capacity to produce 35,000 and 60,000 liters of molasses ethanol per day, respectively. On the other hand, Kamtech logistics in Lira has installed a capacity of 4000 liters of cassava ethanol per day [200]. It is worth noting that the first-generation ethanol can be produced from feedstocks that contain sugar, for example, sugar beet, sugarcane, and molasses. However, it can also be obtained from starch crops such as maize, cassava, banana, and sweet sorghum. Contemplating biogas energy technology has been of interest to a number of researchers in recent years.  Table 11 presents the examples of the previous studies on biogas energy in Uganda.

### 13.2. Challenges and Their Possible Mitigation

The challenges of the development and utilization of nonsolid biomass are quite similar to the previous energy systems. Moreover, the barriers to the wider implementation of biogas as a source of energy have been comprehensively reviewed by previous studies [209–215]. However, some of the challenges include lack of technology and capacity building, high investment costs, and poor country's regulation, policy, and standards. Similarly, the mitigation of these challenges can be adopted from the previously discussed energy systems.

### 13.3. Future Trends

The need for the future development and utilization of nonsolid biomass is so demanding as a strategy to address climate change. The country should expand the existing biogas plants to all villages. Gasification should be studied and adopted in the country because it is the technology that promises clean fuel to mitigate climate change and uses varieties of cheap and locally available feedstocks such as agricultural and agroprocessing residues and MSW [216–218]. Biofuel or biodiesel, ethanol, and methanol production facilities should be developed or expanded since the country is very rich in biomass. There are a lot of underutilized agricultural and agroprocessing residues that need to be converted into useful resources. This has opened new ground for research in bioethanol production. For instance, a study has been conducted on bioethanol production from different matooke peels species [219]. The future clean energy that is going to change the country energy profile with less or no environmental concern is nonsolid biomass [220, 221]. Therefore, the country should plan to forget the traditional use of solid biomass.

For instance, biogas uptake among the community is still below average in most developing countries [222]. This calls for new thinking in biogas dissemination strategy and business model. Search for feedstocks for both biogas and gasification. Codigestion (two feedstocks) of mixed waste is also an attractive area of research, for example, biogas production from livestock manures and slaughterhouse waste [223]. Moreover, a recent study assessed the entrepreneurial potential and feasibility of developing a mobile system for purifying and bottling biogas in portable cylinders for wider society consumption and benefit. This could increase biogas energy supply and access in developing countries [201]. Therefore, extensive study is recommended in this area. Further, smart systems for monitoring the biogas production process are also attracting research today [224].

## 14. Discussions and Recommendations

Human and animal energy sources are still the most utilized energy sources in rural areas of Uganda. However, there is still a gap in its utilization. Unfortunately, many youths are currently unemployed and eventually waste resources on activities such as gambling or betting and leisure. In order to harness the power of humans and animals to the fullest, there is a need for proper technology and tools design. For instance, the mechanization of agriculture is one of the technologies required. By using proper technology and tools, human and animal energy can be sustainably utilized without making them drudgery.

The traditional cooking stoves that use firewood are still dominant within the rural population of Uganda. Overdependence on firewood as the source of energy has resulted in deforestation. Nonetheless, it is still a hurdle for the government to provide cheap fuel like firewood to the rural population in order to reduce the overreliance on firewood. Worst still, charcoal, a processed wood fuel, is being used among the urban people. This makes no difference in terms of deforestation, though it is cleaner than firewood with less or no smoke problem. Technology such as briquetting of agricultural residues and municipal wastes (MSW) can be a better solution to the overutilization of firewood. The use of other energy sources for cooking, such as LPG, biogas, electricity, and ethanol, is paramount important to minimize overdependence on firewood.

Hydropower is the hope and future for Uganda electricity, as nuclear, geothermal, and wind are still paperwork. River Nile is the miracle river for Uganda, where most of the large hydropower stations are constructed. The country's overdependence on hydropower for electricity is indeed not very safe for the future. With so many challenges of hydropower, such as climate change and high investment and maintenance costs, the country will not meet electricity demand in the future. Therefore, Uganda should continue to explore and develop other energy sources for electricity.

Wind energy in Uganda is still a story with so many questions unanswered regarding whether it will be economically feasible to install the wind turbines in the sites identified or not. Wind energy requires a huge amount of land, which is not easy to be given out by the local people, and compensating all these people will require enormous capital investment costs. Nevertheless, a small wind farm can be established to supply the specific settlement without the need to connect to the national grid.

Solar energy from PV cells is currently being adopted by most people in villages. So many places where the hydropower national grid is not reaching are massively depending on solar PV electricity to power their electronic devices, lighting, and so many other uses. However, the application of PV electricity should be expanded, such as cooking, powering agricultural machines, and crop processing.

Geothermal energy in Uganda has not been developed, and it is still more paperwork. There is still much to be done to start harvesting geothermal energy. The competition with many cheaper energy sources such as hydropower and solar will not make geothermal energy come to life any time sooner. However, the country should not look down on geothermal energy because having diverse energy sources is essential for the sustainability of power utilization in the country.

Nuclear energy is the atomic energy of the future for Uganda. Nuclear is being feared and wanted at the same time. With the availability of large deposits of uranium, Uganda stands at the edge of developing nuclear energy in the near future. The only challenge is meeting the 19 nuclear energy infrastructure requirements, such as availability of safety regulation, nuclear waste storage, technologies, and skilled manpower. Despite the hurdle in overcoming the nuclear energy challenges, the country should never give up. This is because nuclear energy is the only energy that will reduce the country's overdependence on hydropower for electricity.

Peat energy is a forgotten but very important energy source that can be harnessed cheaply. Uganda is blessed with large peatlands. Peat is not like coal or petroleum; in fact, it is between renewable and fossil fuel. That means it is not a full fossil fuel or renewable energy source. The utilization of peat will be a great opportunity for Uganda to diversify its energy sources which in turn will improve the overall sustainability of the energy sources in the country.

Coal energy, the black gold, is just a story for a country like Uganda. There are not many studies done on coal, so there is no idea of whether Uganda has a coal deposit or not. Importing coal from other countries such as Tanzania or DRC will be too expensive for the country to economically benefit from such an energy source. Nonetheless, it is better for the country to remain without coal utilization. This is because the utilization of coal comes with so many problems that the country might not handle.

Petroleum energy is still the only single energy system for the transportation industry in Uganda. Petroleum is just full of geopolitics, and thus, the main challenge with its utilization. Uganda being a landlocked country suffered from the importation of petroleum, making the transportation industry unstable in terms of fare. The rise in transport costs is then directly linked to the prices of commodities. The newly crude oil mining at Albertine is now the hope for the country as much as it is being exported for processing. For a country to be self-sufficient in the oil field, it has to develop its own processing plants with all the technologies and human skills required. This is the only way the country can be independent of itself.

Lastly, nonsolid biomass such as hydrogen, biofuel, biogas, ethanol, and methanol are very clean and renewable energy sources that the country should bet big on them. For instance, biogas can be an alternative to firewood for cooking as well as LPG. Gasification of the agricultural residues and MSW is also potential technology that can be used for cooking and transportation (hydrogen gas). So far so good; the country is trying to develop and install biogas and ethanol plants. However, the country should also look at biomass gasification or even cogasification with MSW. Gasification of MSW offers more potential than energy recovery from MSW incineration [225]. Cogasification is vitally important because it is the technology that will solve both energy and environmental problem. In these regards, researches are required to understand the suitability of biomass gasification in the country, and also studies are needed to gain insight into the cogasification process of several feedstock combinations.

## 15. Conclusion

The present study successfully reviewed energy systems in Uganda. Human and animal energy is the forgotten renewable energy that needs to be harnessed sustainably to avoid drudgery. Hydropower is still the alpha and omega for the country as far as electric power is concerned. There is a need for the country to diversify its energy systems for electricity generation. Thus, the only hope for the country future electricity generation is from atomic energy (nuclear energy). Though it is not a renewable energy source, nuclear energy will be very vital to minimize the country overdependence on hydropower. Many energy systems are still underdeveloped or underutilized. Geothermal, wind, peat, and nuclear energy systems are still in an early stage of development and are not utilized. However, there is a plan for the development to reach the utilization stage in the near future. Solar energy and biogas energy are the most studied energy systems in Uganda in the academic literature. Thermochemical processing of biomass and nonbiomass wastes into syngas or hydrogen is becoming a more attractive area of research today, as there is a rising need to produce clean hydrogen with reduced production and raw material cost. Bioethanol energy is one of the adopted nonsolid biomass energies in Uganda; however, its application for transportation in the country is still limited compared with other developed nations. The present study has not covered in detail the technologies used for harvesting or harnessing these energy systems. Therefore, further study is required to review the technologies behind the energy systems utilization, and the impact of these technologies on energy development and utilization should be investigated. In addition, several energy systems were not covered in this paper. Particularly, the tidal, wave, battery, fuel cells, hybrid, and gravity energy storage systems need to be investigated for their utilization and development in the country.

## Figures and Tables

**Table 1:** Summary of previous review papers on energy systems.

S/N	Authors/year	Title	Review approach	Study area	Purpose
1	Wassie and Adaramola, 2019 [53]	Potential environmental impacts of small-scale renewable energy technologies in east Africa: a systematic review of the evidence	Systematic	East Africa	Provide a comprehensive review and analysis of the potential impacts of small-scale renewable energy technologies (SRETs) in reducing deforestation, forest degradation, and carbon emissions in the eastern African region.
2	Ahmad and Zhang, 2020 [54]	A critical review of comparative global historical energy consumption and future demand: the story told so far	Traditional narrative	OECD, G7, BRICS, Europe, EU, (CIS), NA, LA, USA, Asia, the Pacific, and ME	Present a critical combined energy analysis of demand in developed/developing countries, including the load requirements of the various business sectors.
3	Mutumba et al., 2021 [55]	A survey of literature on energy consumption and economic growth	Systematic (meta-analysis)	—	Investigate stationarity, cointegration, and direction of causality between energy consumption and economic growth.
4	Okumu et al., 2021 [56]	A review of water-forest-energy-food security nexus data and assessment of studies in east Africa	Systematic	East Africa	Provide a critical analysis and synthesis of the baseline and trends of the forest-water-food-energy security nexus in East Africa.
5	Baidhe et al., 2021 [57]	Unearthing the potential of solid waste generated along the pineapple drying process line in Uganda: a review	Traditional narrative	Uganda	Review the potential use of novel scientific and technical methods for pineapple waste (peelings, crown, core, and culled pineapple) management at dried fruit processing facilities in Uganda.
6	Falchetta et al., 2019 [58]	Hydropower dependency and climate change in sub-Saharan Africa: a nexus framework and evidence-based review	Systematic (PRISMA)	Sub-Saharan Africa	Provide supporting evidence on past trends and current pathways of power mix diversification, drought incidence, and climate change.
7	Carlson et al., 2015 [59]	Petroleum pipeline explosions in sub-Saharan Africa: a comprehensive systematic review of the academic and lay literature	Systematic (PRISMA)	Sub-Saharan Africa	Evaluate both the academic and lay literature on petroleum pipeline explosions in sub-Saharan Africa.
8	Hansen et al., 2015 [60]	Review of solar PV policies, interventions and diffusion in east Africa	Traditional narrative	East Africa	Identify the key factors put forward in the literature to explain differences in the diffusion of SHS in these three countries.
9	Mwirigi et al., 2014 [61]	Socioeconomic hurdles to widespread adoption of small-scale biogas digesters in sub-Saharan Africa: a review	Traditional narrative	Sub-Saharan Africa	Examine the socioeconomic constraints to adoption of biogas in sub-Saharan Africa and explore factors that could enhance adoption of the technology.
10	Okot, 2013 [62]	Review of small hydropower technology	Traditional narrative	—	Give a review of small hydropower technology.
11	Ojong, 2021 [63]	The rise of solar home systems in sub-Saharan Africa: examining gender, class, and sustainability	Systematic	Sub-Saharan Africa	Investigate how the forms of social difference shape the adoption of SHSs in sub-Saharan Africa.

Commonwealth of Independent States (CIS), North America (NA), Latin America (LA), the United States of America (USA), Asia, the Pacific, and the Middle East (ME).

**Table 2:** Comparison of load carrying capacity and distances for hand-and-foot-operated devices.

Transport mode	Typical load (kg)	Average speed (km/h)	Daily range (km)	Transport capacity (t km/h)
Human carrying	30	4-5	15–20	0.12
Wheelbarrow	90	3-4	5-6	0.35
Handcart (1 person)	200	3-4	10–12	0.80
Cycle with carrier	40	12	40–50	0.48
Cycle trailer	125	10	30–40	1.25
Donkey cart (1 animal)	300	3-4	20	1.10
Ox cart (2 animals)	900	3-4	20	3.20

Source: adapted from Dennis [70].

**Table 3:** Operational large hydropower plants.

S/N	Power stations	Community (district)	River	Capacity (MW)	Year completed
1	Nalubaale	Buikwe	Nile	180	1954
2	Kiira	Jinja	Nile	200	2000
3	Bujagali	Buikwe	Nile	250	2012
4	Mahoma	Kabarole	Mahoma	30	2018
5	Isimba	Kamuli	Nile	183	2019
6	Achwa 2	Gulu	Achwa	41	2019
7	Achwa 1	Gulu	Achwa	42	2021
	Total			926	

Source: adapted from Tumwesigye et al. [32].

**Table 4:** Operational small hydropower plants.

S/N	Power stations	District	River	Capacity (MW)	Year done
1	Mubuku 1	Kasese	Mubuku	5.0	1956
2	Mubuku 3	Kasese	Mubuku	10.0	2009
3	Bugoye	Kasese	Mubuku	13.0	2009
4	Kisiizi	Rukungiri	Kisiizi	0.4	2009
5	Echo power-Ishasha	Kanungu	Ishasha	6.6	2011
6	Africa EMS-Mpanga	Kamwenge	Mpanga	18.0	2011
7	Nyagak 1	Zombo	Nyangak	3.5	2012
8	Kabalega	Hoima	Wambabya	9.0	2013
9	Bwindi community	Kanungu	Munyaga	0.1	2014
10	Siti 1	Bukwo	Siti	6.1	2017
11	Muvumbe	Kabale	Maziba	6.5	2017
12	Rwimi	Bunyangabu	Rwimi	5.6	2017
13	Siti 2	Bukwo	Siti	16.5	2017
14	Gwera-Luzira	Moyo	Amoa	6.1	2017
15	Nyamwamba 1	Kasese	Nyamwamba	9.2	2018
16	Nkusi	Kasese	Nkusi	9.6	2018
17	Lubilia	Hoima	Lubilia	5.4	2018
18	Nyamagasani II	Kasese	Nyamagasani	6.0	2019
19	Kyambura	Rubirizi	Kyambura	7.6	2019
20	Ndugutu	Bundibugyo	Ndugutu	5.9	2019
21	Sindila	Bundibugyo	Sindila	5.3	2019
22	Timex Bukinda	Kibale	Nkusi	6.5	2020
23	Kikagati	Isingiro	Kagera	14.0	2021
24	Kakaka	Kasese	Rwimi	4.6	2021
25	Nyamagasani I	Kasese	Nyamagasani	15.0	2021
	Total			195.5	

Source: adapted from Tumwesigye et al. [32] and ERA [101].

**Table 5:** Proposed and under construction large hydropower plant [118, 119].

S/N	Power stations	Community (district)	River	Capacity (MW)	Year to be completed
1	Achwa 3	Pader	Achwa	135	2022
2	Karuma	Kiryandongo	Nile	600	2023
3	Ayago	Nwoya	Nile	880	2025
4	Agbinika	Yumbe	Tochi	20	2025
5	Nshungyezi	Isingiro	Kagera	39.0	2025
6	Kiiba	Kiryandongo and Nwoya	Nile	400	WIP
7	Oriang	Kiryandongo and Nwoya	Nile	392	WIP
8	Muzizi	Kibaale	Muzizi	48	WIP
	Total			2514	

**Table 6:** Small hydropower plants under construction.

S/N	Power stations	Districts	River	Capacity (MW)	Year
1	Nengo Bridge	Rukungiri	Mirera	6.7	2022
2	Nyagak 3	Zombo	Nyagak	5.6	2022
3	Nyamwabwa 2	Kasese	Nyamwabwa	7.8	2022
4	Muyembe	Kapchorwa		6.9	2022
5	Nyagak 2	Zombo	Nyagak	5.0	2023
	Total			32.0	

Source: adapted from ERA [118].

**Table 7:** Proposed small hydropower plants.

S/N	Power stations	District	River	Capacity (MW)	Year
1	Nsongi	Bunyangabu	Nsongya	7.0	WIP
2	Kiraboha	Kasese	Rwimi	5.0	WIP
3	Latoro	Nwoya	Achwa	4.2	WIP
4	Buwangani	Manafwa	Manafwa	7.0	WIP
5	Nyakinengo	Kanungu	Nchwera	5.2	WIP
6	Lower Achwa	Lamwo and Amuru	Achwa	17.4	WIP
7	Awera	Pader	Achwa	18.0	WIP
8	Okollo	Arua	Ora	5.0	WIP
9	Rwembya	Kasese	Rwembya	0.4	WIP
10	Lwakhakha	Namisidwa	Lwakhakha	6.7	WIP
11	Senok Atari 1	Kapchorwa	Atari	3.3	WIP
12	Kabeywa 1	Bulambuli	Mbigi	6.5	WIP
13	Kabeywa 2	Kapchorwa	Sirimityo	2.0	WIP
14	Sironko	Sironko	Sironko	7.0	WIP
15	Nyabuhuka-Mujunju	Bunyangabu	Nsongya	3.2	WIP
16	Simu	Bulambuli	Simu	9.5	WIP
17	Sisi	Bulambuli	Sisi	7.0	WIP
18	Kigwabya	Kagadi	Nkusi	4.2	WIP
19	Warugo	Bushenyi	Warugo	0.5	WIP
20	Igassa	Bunyangabu	Igassa	0.3	WIP
21	Tokwe	Bundibugyo	Tokwe	0.3	WIP
22	Nyahuka	Bunyibugyo	Nyahuka	0.7	WIP
23	Nsongya	Bunyangabu	Nsogya	0.7	WIP
24	Katooke	Kasese	Nyabyayi	0.3	WIP
25	Nchwera	Mitooma	Nchwera	0.5	WIP
26	Hoima	Hoima	Hoimo	3.3	WIP
27	Kabasanja	Kabarole	Wamikia	0.4	WIP
	Total			125.6	

WIP: work in progress; source: adapted from ERA [118].

**Table 8:** Small hydropower plant available for development.

S/N	Power stations	District	Capacity (MW)	Status
1	Ela	Arua	1.5	No studies
2	Ririma	Kapchorwa	1.5	No studies
3	Rwigo	Bundibugyo	0.48	No studies
4	Nyarwodo	Nebbi	0.4	No studies
5	Agoi	Arua	0.35	No studies
6	Kitumba	Kabale	0.2	No studies
7	Tokwe	Bundibugyo	0.4	Preliminary technical studies carried out under AERDP by MEMD
8	Amua	Moyo	0.18	No studies
9	Ngiti	Bundibugyo	0.15	Preliminary technical studies carried out under AERDP by MEMD
10	Leya	Moyo	0.15	No studies
11	Nyakibale	Rum Rukungiri	0.1	No studies
12	Miria Adua	Arua	0.1	No studies
13	Manafwa	Mbale	0.15	Preliminary technical studies carried out under AERDP by MEMD
	Total		5.66	

Source: adapted from ERA [118].

**Table 9:** Summary of literature on solar energy in Uganda.

S/N	Author/year	Title	Purpose	Research design	Key finding
1	Oloya et al., 2021 [130]	Techno-economic assessment of 10 MW centralised grid-tied solar photovoltaic system in Uganda	Performance analysis of a 10 MW solar photovoltaic plant installed in Soroti city, in eastern Uganda (latitude 1°N, longitude 33°E)	IEC standard 61724-1 and a combination of dynamic and static capital investment methods	Average annual energy generation by the plant is 16702 MWh, and the specific energy output is 1670.2 kWh/kW
2	Groenewoudt et al., 2020 [131]	From fake solar to full service: an empirical analysis of the solar home systems market in Uganda	Analyze the role of product quality in the transition to cleaner energy technologies in developing countries	Qualitative (market survey)	Neither high-quality nor low-quality solar products offer a win-win situation if we are to achieve “access to affordable, reliable, sustainable, and modern energy for all” (SDG 7)
3	Aarakit et al., 2021 [132]	Adoption of solar photovoltaic systems in households: evidence from Uganda	Analyze factors influencing households' choice of solar PV system	Mixed methods	The determinants of adoption, as well as the type of solar PV adopted, are heterogeneous. Rural residence, income, and type of house are significant drivers of solar PV type adopted
4	Thadani and Go, 2021 [133]	Integration of solar energy into low-cost housing for sustainable development: case study in developing countries	Integrate clean energy into a low-cost housing development for sustainable cities in Uganda and Indonesia	Quantitative	The levelized cost of electricity (LCOE) with and without an optimizer ranged from $0.25/kWh to $0.36/kWh for Uganda
5	Mukisa et al., 2019 [134]	Feasibility assessment of grid-tied rooftop solar photovoltaic systems for industrial sector application in Uganda	Evaluate the feasibility of implementing grid-tied rooftop solar PV systems in the industrial sector in Uganda	Quantitative	Possible annual energy yield in the range of 1046 kWh/kW–1344 kWh/kW for all roof orientations and roof tilt angles in the range of 0°–45°
6	Bhamidipati et al., 2019 [135]	Agency in transition: the role of transnational actors in the development of the off-grid solar PV regime in Uganda	Investigate the role of transnational actors in the development of the off-grid solar PV regime in Uganda, from the early 1980s to 2017	Qualitative	Demonstrate empirically the highly transnational nature of regime development
7	Eder et al., 2015 [136]	Mini-grids and renewable energy in rural Africa: how diffusion theory explains adoption of electricity in Uganda	Analyzes the factors that influence the adoption of renewable electricity from individual households' perspectives	Qualitative case study	There is an emphasis on the relative advantages of the new technology. Second, there are economic requirements regarding a viable financial system for adopters, especially in such a low-income market

**Table 10:** Prospective geothermal sites in Uganda [118].

District	Site	Temperature	Remarks on status
Kasese	Katwe-Kikorongo	Surface temperature: 71°C Inferred reservoir temperature: 150–230°C	There is sufficient information for the development of a geothermal energy programme on the Katwe-Kikorongo site, which has been selected for drilling the first geothermal well in Uganda. The site has occurrence of a medium-to-high-temperature resource.

Bundibugyo	Buranga	Surface temperature: 97°C Inferred reservoir temperature: 120–150°C	Nyansimbe and Mumbuga in Buranga (Sempaya valley) have the highest surface heat output among the thermal prospects considered and provided sufficient information for the development of geothermal energy programme.

Hoima	Kibiro	Surface temp: 84°C Inferred reservoir temp: 200°C and above	Kibiro site has moderate surface heat output among the thermal prospects considered and provided sufficient information for the development of the geothermal energy programme.

**Table 11:** A summary of previous studies on biogas energy in Uganda.

S/N	Author/year/reference	Title	Purpose	Research design/method	Key finding
1	Namugenyi et al., 2022 [201]	Realising the transition to bioenergy: integrating entrepreneurial business models into the biogas socio-technical system in Uganda	Assess the entrepreneurial potential and feasibility of developing a mobile system for purifying and bottling biogas in portable cylinders for wider society consumption and benefit	A multimethod approach comprising semistructured interviews, nonparticipant observation, document analysis, and a feasibility study	Existing research has neglected the entrepreneurial potential in biogas energy that could increase energy supply and access in developing countries
2	Clemens et al., 2018 [202]	Africa biogas partnership program: a review of clean cooking implementation through market development in east Africa	Analyze the Africa biogas partnership program in Kenya, Tanzania, and Uganda	Literature survey (RE-AIM framework) and interview	Between 2009 and 2017, over 27,000 households installed a biodigester, half of them in Kenya. Biodigester development hubs numbers: Kenya (22); Uganda (5); Tanzania (7)
3	Tumusiime et al., 2019 [203]	Long-life performance of biogas systems for productive applications: the role of R&D and policy Edmund	Evaluate the performance of productive biogas installations with the aim of determining the root cause of this poor performance	Mixed research design (both interviews and experimental tests)	50% of productive biogas installations failed within two years after their commissioning due to logistical and technological challenges. Insufficient R&D in the biogas sector is suggested to be the lead cause of such poor performance
4	Ogwang et al., 2021 [204]	Characterization of biogas digestate for solid biofuel production in Uganda	Investigate the suitability of digestate from anaerobic digestion of cow dung, pig dung, and human waste feedstock as a solid fuel for thermal applications	Experimental	The briquettes from the biogas digestate demonstrate potential for domestic thermal applications in Uganda
5	Nalunga et al., 2019 [205]	The dynamics of household labor allocation to biogas production, farm and nonfarm activities in central Uganda	Assess the factors influencing labor allocation of biogas production within farm households	Field survey (qualitative)	Household labor should be critically analyzed before investing in biogas digesters to in- crease the success of the technology
6	Lwiza et al., 2017 [206]	Dis-adoption of household biogas technologies in central Uganda Florence	Analyze dis-adoption of biogas technologies in central Uganda	Cross-sectional survey	An increase in the family size, the number of cattle, the number of pigs, and the age of the household head reduced the likelihood of biogas technology dis-adoption
7	Walekhwa et al., 2014 [207]	Economic viability of biogas energy production from family-sized digesters in Uganda	Assess economic viability of biogas energy production in Uganda	Field survey	Biogas energy production is economically viable with a payback period of 1.17, 1.08, and 1.01 years for 8 m^3^, 12 m^3^, and 16 m^3^ biogas plants, respectively
8	Kabyanga et al., 2018 [208]	Are smallholder farmers willing to pay for a flexible balloon biogas digester? Evidence from a case study in Uganda Moris	Investigate the farmers' willingness to pay for a flexible balloon biogas digester	Case study with field survey	The majority of surveyed households showed their willingness to pay, but an average household's maximum WTP (US$52) was ten times less than the actual cost of an imported flexible balloon digester unit (US$512)

## Data Availability

Research data underlying the findings of the study are included in the paper.

## References

[1] Mohammed Y. S., Mustafa M. W., Bashir N. (2013). Status of renewable energy consumption and developmental challenges in sub-Sahara Africa. *Renewable and Sustainable Energy Reviews*.

[2] Munro P. G., Bartlett A. (2019). Energy bricolage in northern Uganda: rethinking energy geographies in sub-Saharan Africa. *Energy Research & Social Science*.

[3] Miller R. L., Ulfstjerne M. A. (2020). Trees, tensions, and transactional communities: problematizing frameworks for energy poverty alleviation in the rhino camp refugee settlement, Uganda. *Energy Research & Social Science*.

[4] Ssennono V. F., Ntayi J. M., Buyinza F., Wasswa F., Aarakit S. M., Mukiza C. N. (2021). Energy poverty in Uganda: evidence from a multidimensional approach. *Energy Economics*.

[5] Murphy P. M., Twaha S., Murphy I. S. (2014). Analysis of the cost of reliable electricity: a new method for analyzing grid connected solar, diesel and hybrid distributed electricity systems considering an unreliable electric grid, with examples in Uganda. *Energy*.

[6] Ram M., Bogdanov D., Aghahosseini A. (2022). Global energy transition to 100% renewables by 2050: not fiction, but much needed impetus for developing economies to leapfrog into a sustainable future. *Energy*.

[7] Emblemsvåg J. (2022). Wind energy is not sustainable when balanced by fossil energy. *Applied Energy*.

[8] Probst B., Westermann L., Anadón L. D., Kontoleon A. (2021). Leveraging private investment to expand renewable power generation: evidence on financial additionality and productivity gains from Uganda. *World Development*.

[9] Sun Y., Hao Q., Cui C. (2022). Emission accounting and drivers in east African countries. *Applied Energy*.

[10] Zhang F., Tang T., Su J., Huang K. (2020). Inter-sector network and clean energy innovation: evidence from the wind power sector. *Journal of Cleaner Production*.

[11] Maji I. K. (2019). Impact of clean energy and inclusive development on CO_2_ emissions in sub-Saharan Africa. *Journal of Cleaner Production*.

[12] Leonard A., Ahsan A., Charbonnier F., Hirmer S. (2022). The resource curse in renewable energy: a framework for risk assessment. *Energy Strategy Reviews*.

[13] Riti J. S., Riti M. K. J., Oji-Okoro I. (2022). Renewable energy consumption in sub-Saharan Africa (SSA): implications on economic and environmental sustainability. *Current Research in Environmental Sustainability*.

[14] Barasa M., Bogdanov D., Oyewo A. S., Breyer C. (2018). A cost optimal resolution for sub-Saharan Africa powered by 100% renewables in 2030. *Renewable and Sustainable Energy Reviews*.

[15] Amir M., Khan S. Z. (2022). Assessment of renewable energy: status, challenges, COVID-19 impacts, opportunities, and sustainable energy solutions in Africa. *Energy and Built Environment*.

[16] de la Rue du Can S., Pudleiner D., Pielli K. (2018). Energy efficiency as a means to expand energy access: a Uganda roadmap. *Energy Policy*.

[17] Basudde P. (2020). Promoting the transfer and development of climate-smart energy technologies in Uganda. *Encyclopedia of the World’s Biomes*.

[18] Trotter P. A., Maconachie R. (2018). Populism, post-truth politics and the failure to deceive the public in Uganda’s energy debate. *Energy Research & Social Science*.

[19] Adu D., Jianguo D., Darko R. O., Boamah K. B., A-Boateng E. (2020). Investigating the state of renewable energy and concept of pump as turbine for energy generation development. *Energy Reports*.

[20] Escribano G. (2021). *Beyond Energy Independence*.

[21] De Angelis P., Tuninetti M., Bergamasco L. (2021). Data-driven appraisal of renewable energy potentials for sustainable freshwater production in Africa. *Renewable and Sustainable Energy Reviews*.

[22] Mukoro V., Gallego-Schmid A., Sharmina M. (2021). Life cycle assessment of renewable energy in Africa. *Sustainable Production and Consumption*.

[23] Ambole A., Musango J. K., Buyana K. (2019). Mediating household energy transitions through co-design in urban Kenya, Uganda and South Africa. *Energy Research & Social Science*.

[24] Okoboi G., Mawejje J. (2016). Electricity peak demand in Uganda: insights and foresight. *Energy, Sustainability and Society*.

[25] Habtu D., Ahlgren E. O., Bekele G. (2021). Long-term evolution of energy and electricity demand forecasting: the case of Ethiopia. *Energy Strategy Reviews*.

[26] Joint Research Centre (2020). *Status of Geothermal Industry in East African Countries*.

[27] Wabukala B. M., Otim J., Mubiinzi G., Adaramola M. S. (2021). Assessing wind energy development in Uganda: opportunities and challenges. *Wind Engineering*.

[28] ERA (2020). Electricity supply industry performance report for the year 2019. https://www.era.go.ug/index.php/resource-centre/publications/reports/561-electricity-supply-industry-performance-report-2019/download.

[29] Duguma L. A., Kay S., Okia C. A. (2021). The potentials of technology complementarity to address energy poverty in refugee hosting landscapes in Uganda. *Energy, Ecology and Environment*.

[30] ERA (2021). *Schedule of End-User Tariffs Applicable for the Supply of Electricity by Umeme limited for the Third Quarter of the Year 2021*.

[31] Kakumba M. R. (2021). Despite hydropower surplus, most Ugandans report lack of electricity. https://bit.ly/3wXK9GT.

[32] Tumwesigye R., Twebaze P., Makuregye N., Muyambi E. (2011). Key issues in Uganda’s energy sector. https://pubs.iied.org/sites/default/files/pdfs/migrate/16030IIED.pdf.

[33] Van Der Ven M. J. (2020). *An Overview of Recent Developments and the Current State of the Ugandan Energy Sector*.

[34] Ang C., John N., Oludhe C., Chitedze I. (2020). Heliyon the role of diversity, reserve margin and system structure on retail electricity tariffs in Kenya. *Heliyon*.

[35] Olleik M., Auer H., Nasr R. (2021). A petroleum upstream production sharing contract with investments in renewable energy: the case of Lebanon. *Energy Policy*.

[36] Ai H., Xiong S., Li K., Jia P. (2020). Electricity price and industrial green productivity: does the “low-electricity price trap” exist?. *Energy*.

[37] Mwaura F. M. (2012). Adopting electricity prepayment billing system to reduce non-technical energy losses in Uganda: lesson from Rwanda. *Utilities Policy*.

[38] Hirmer S., Guthrie P. (2017). The benefits of energy appliances in the off-grid energy sector based on seven off-grid initiatives in rural Uganda. *Renewable and Sustainable Energy Reviews*.

[39] Hirmer S., Guthrie P. (2016). Identifying the needs of communities in rural Uganda: a method for determining the “user-perceived value” of rural electrification initiatives. *Renewable and Sustainable Energy Reviews*.

[40] Bhamidipati P. L., Haselip J., Elmer Hansen U. (2019). How do energy policies accelerate sustainable transitions? Unpacking the policy transfer process in the case of GETFiT Uganda. *Energy Policy*.

[41] Monyei C. G., Akpeji K. O., Oladeji O. (2022). Regional cooperation for mitigating energy poverty in sub-Saharan Africa: a context-based approach through the tripartite lenses of access, sufficiency, and mobility. *Renewable and Sustainable Energy Reviews*.

[42] Purdon M. (2015). Opening the black box of carbon finance “additionality”: the political economy of carbon finance effectiveness across Tanzania, Uganda, and Moldova. *World Development*.

[43] Athukorala W., Wilson C., Managi S., Karunarathna M. (2019). Household demand for electricity: the role of market distortions and prices in competition policy. *Energy Policy*.

[44] Balarama H., Islam A., Kim J. S., Wang L. C. (2020). Price elasticities of residential electricity demand: estimates from household panel data in Bangladesh. *Energy Economics*.

[45] Deng C., Jiang Z., Sun C. (2018). Estimating the efficiency and impacts of petroleum product pricing reforms in China. *Sustainability*.

[46] Güler H., Tedgren C. (2009). *Establishing the Optimal Tariff in Rural Electricity Distribution Networks: A Case Study in Uganda*.

[47] The Oxford Institute for Energy Studies (2015). *Oil in Uganda: Hard Bargaining and Complex Politics in East Africa*.

[48] ESMAP (2009). *Petroleum Product Markets in Sub-Saharan Africa*.

[49] Sexsmith F. (2010). *Petroleum Markets in Sub-Saharan Africa Petroleum Markets in Sub-Saharan Africa Analysis*.

[50] OECD (2020). *The Impact of Coronavirus (COVID-19) and the Global Oil Price Shock on the Fiscal Position of Oil-Exporting Developing Countries*.

[51] Raghoo P., Surroop D. (2020). Price and income elasticities of oil demand in Mauritius: an empirical analysis using co-integration method. *Energy Policy*.

[52] Twaha S., Ramli M. A. M., Murphy P. M., Mukhtiar M. U., Nsamba H. K. (2016). Renewable based distributed generation in Uganda: resource potential and status of exploitation. *Renewable and Sustainable Energy Reviews*.

[53] Wassie Y. T., Adaramola M. S. (2019). Potential environmental impacts of small-scale renewable energy technologies in east Africa: a systematic review of the evidence. *Renewable and Sustainable Energy Reviews*.

[54] Ahmad T., Zhang D. (2020). A critical review of comparative global historical energy consumption and future demand: the story told so far. *Energy Reports*.

[55] Mutumba G. S., Odongo T., Okurut N. F., Bagire V. (2021). A survey of literature on energy consumption and economic growth. *Energy Reports*.

[56] Okumu B., Kehbila A. G., Osano P. (2021). A review of water-forest-energy-food security nexus data and assessment of studies in east Africa. *Current Research in Environmental Sustainability*.

[57] Baidhe E., Kigozi J., Mukisa I., Muyanja C., Namubiru L., Kitarikawe B. (2021). Unearthing the potential of solid waste generated along the pineapple drying process line in Uganda: a review. *Environmental Challenges*.

[58] Falchetta G., Gernaat D. E. H. J., Hunt J., Sterl S. (2019). Hydropower dependency and climate change in sub-Saharan Africa: a nexus framework and evidence-based review. *Journal of Cleaner Production*.

[59] Carlson L. C., Rogers T. T., Kamara T. B. (2015). Petroleum pipeline explosions in sub-Saharan Africa: a comprehensive systematic review of the academic and lay literature. *Burns*.

[60] Hansen U. E., Pedersen M. B., Nygaard I. (2015). Review of solar PV policies, interventions and diffusion in east Africa. *Renewable and Sustainable Energy Reviews*.

[61] Mwirigi J., Balana B. B., Mugisha J. (2014). Socio-economic hurdles to widespread adoption of small-scale biogas digesters in sub-Saharan Africa: a review. *Biomass and Bioenergy*.

[62] Okot D. K. (2013). Review of small hydropower technology. *Renewable and Sustainable Energy Reviews*.

[63] Ojong N. (2021). The rise of solar home systems in sub-Saharan Africa: examining gender, class, and sustainability. *Energy Research & Social Science*.

[64] Bongomin O., Nganyi E. O., Abswaidi M. R., Hitiyise E., Tumusiime G. (2020). Sustainable and dynamic competitiveness towards technological leadership of industry 4.0: implications for east african community. *Journal of Engineering*.

[65] Bongomin O., Ocen G., Oyondi Nganyi E., Musinguzi A., Omara T. (2020). Exponential disruptive technologies and the required skills of industry 4.0. *Journal of Engineering*.

[66] Bongomin O., Yemane A., Kembabazi B. (2020). Industry 4.0 disruption and its neologisms in major industrial sectors: a state of the art. *Journal of Engineering*.

[67] Fuller R. J., Aye L. (2012). Human and animal power—the forgotten renewables. *Renewable Energy*.

[68] Mota-Rojas D., Braghieri A., Alvarez-Macias A. (2021). The use of draught animals in rural labour. *Animals*.

[69] Mechtenberg A. R., Borchers K., Miyingo E. W. (2012). Human power (HP) as a viable electricity portfolio option below 20 W/Capita. *Energy for Sustainable Development*.

[70] Dennis R. (1999). Meeting the challenge of animal-based transport. *A Resource Book of Animal Traction Network for Eastern and Southern Africa*.

[71] Netam A., Jaiswal P. (2018). Role of animal power in the field of agriculture. *International Journal of Avian & Wildlife Biology*.

[72] FAO (2010). Draught animal power: an overview. https://www.fao.org/ag/ags/agse/chapterps1/chapterps1-e.htm.

[73] Shirley R., Liu Y., Kakande J., Kagarura M. (2021). Identifying high-priority impact areas for electricity service to farmlands in Uganda through geospatial mapping. *Journal of Agriculture and Food Research*.

[74] Nussbaumer P., Bazilian M., Modi V. (2012). Measuring energy poverty: focusing on what matters. *Renewable and Sustainable Energy Reviews*.

[75] Tumwesige V., Okello G., Semple S., Smith J. (2017). Impact of partial fuel switch on household air pollutants in sub-Sahara Africa. *Environmental Pollution*.

[76] Jagger P., Shively G. (2014). Land use change, fuel use and respiratory health in Uganda. *Energy Policy*.

[77] Etchie A. T., Etchie T. O., Elemile O. O. (2020). Burn to kill: wood ash a silent killer in Africa. *Science of the Total Environment*.

[78] Lea-Langton A. R., Spracklen D., Arnold S. (2019). PAH emissions from an African cookstove. *Journal of the Energy Institute*.

[79] Quinn A. K., Bruce N., Puzzolo E. (2018). An analysis of efforts to scale up clean household energy for cooking around the world. *Energy for Sustainable Development*.

[80] Coker E., Katamba A., Kizito S., Eskenazi B., Davis J. L. (2020). Household air pollution profiles associated with persistent childhood cough in urban Uganda. *Environment International*.

[81] Lemma B., Ararso K., Evangelista P. H. (2021). Attitude towards biogas technology, use and prospects for greenhouse gas emission reduction in southern Ethiopia. *Journal of Cleaner Production*.

[82] Rose J., Bensch G., Munyehirwe A., Peters J. (2022). The forgotten coal: charcoal demand in sub-Saharan Africa. *World Development Perspectives*.

[83] Jagger P., Kittner N. (2017). Deforestation and biomass fuel dynamics in Uganda. *Biomass and Bioenergy*.

[84] Twongyirwe R., Bithell M., Richards K. S. (2018). Revisiting the drivers of deforestation in the tropics: insights from local and key informant perceptions in western Uganda. *Journal of Rural Studies*.

[85] Mainimo E. N., Okello D. M., Mambo W., Mugonola B. (2022). Drivers of household demand for cooking energy: a case of Central Uganda. *Heliyon*.

[86] Sassen M., Sheil D., Giller K. E. (2015). Fuelwood collection and its impacts on a protected tropical mountain forest in Uganda. *Forest Ecology and Management*.

[87] Leary J., Leach M., Batchelor S., Scott N., Brown E. (2021). Battery-supported eCooking: a transformative opportunity for 2.6 billion people who still cook with biomass. *Energy Policy*.

[88] Lee L. Y. T. (2013). Household energy mix in Uganda. *Energy Economics*.

[89] Kulindwa Y. J., Lokina R., Ahlgren E. O. (2018). Driving forces for households’ adoption of improved cooking stoves in rural Tanzania. *Energy Strategy Reviews*.

[90] Mwaura F., Okoboi G., Ahaibwe G. (2014). *Determinants of Household’s Choice of Cooking Energy in Uganda*.

[91] Bush G., Nampindo S., Aguti C. (2003). *The Value of Uganda’s Forests: A Livelihoods and Ecosystems Approach*.

[92] Gustavsson M., Broad O., Hankins M., Sosis K. (2015). Energy report for Uganda: a 100% renewable energy future by 2050. https://sun-connect.org/document/energy-report-for-uganda-a-100-renewable-energy-future-by-2050/.

[93] Culver L. C. (2017). *Energy Poverty: What You Measure Matters*.

[94] Bagire V., Wafler M., Rieck C. (2021). Waste as business: emerging Ugandan micro- and small-sized businesses in resource recovery and safe reuse. *Journal of Environmental Management*.

[95] Lietaer S., Zaccai E., Verbist B. (2019). Making cooking champions: perceptions of local actors on private sector development in Uganda. *Environmental Development*.

[96] Abolhosseini S., Heshmati A., Altmann J. (2014). *A Review of Renewable Energy Supply and Energy Efficiency Technologies*.

[97] Breeze P. (2019). Hydropower. *Power Generation Technologies*.

[98] Paish O. (2002). Small hydro power: technology and current status. *Renewable and Sustainable Energy Reviews*.

[99] Kavuma C., Sandoval D., Dieu H. K. J. d., De Dieu J. (2021). Analysis of power generating plants and substations for increased Uganda’s electricity grid access. *AIMS Energy*.

[100] Meyer R., Eberhard A., Gratwick K. (2018). Uganda’s power sector reform: there and back again?. *Energy for Sustainable Development*.

[101] ERA (2015). *Performance Report of Authority for the Period of 2010–2015*.

[102] Agarwal S. S., Kansal M. L. (2020). Risk based initial cost assessment while planning a hydropower project. *Energy Strategy Reviews*.

[103] Larosa F., Rickman J., Ameli N. (2022). Finding the right partners? Examining inequalities in the global investment landscape of hydropower. *Global Environmental Change*.

[104] IEA ETSAP (2010). Hydropower-technology brief E06. https://iea-etsap.org/E-TechDS/PDF/E06-hydropower-GS-gct_ADfina_gs.pdf.

[105] Fashina A., Mundu M., Akiyode O., Abdullah L., Sanni D., Ounyesiga L. (2018). The drivers and barriers of renewable energy applications and development in Uganda: a review. *Clean Technology*.

[106] Bahati H. K., Ogenrwoth A., Sempewo J. I. (2021). Quantifying the potential impacts of land-use and climate change on hydropower reliability of Muzizi hydropower plant, Uganda. *Journal of Water and Climate Change*.

[107] Colenbrander S., Lovett J., Abbo M. S., Msigwa C., M’Passi-Mabiala B., Opoku R. (2015). Renewable energy doctoral programmes in sub-Saharan Africa: a preliminary assessment of common capacity deficits and emerging capacity-building strategies. *Energy Research & Social Science*.

[108] Sovacool B. K., Hess D. J., Cantoni R. (2022). Conflicted transitions: exploring the actors, tactics, and outcomes of social opposition against energy infrastructure. *Global Environmental Change*.

[109] Kimbowa G., Mourad K. A. (2019). Assessing the Bujagali hydropower project in Uganda. https://lupinepublishers.com/ocean-journal/pdf/MAOPS.MS.ID.000141.pdf.

[110] Jansen P., Kugonza R. (2019). *Like Fish on Land: The Impacts of Hydroelectric Power Projects on Resettled Communities in Uganda and Laos*.

[111] Turner S. W. D., Hejazi M., Kim S. H., Clarke L., Edmonds J. (2017). Climate impacts on hydropower and consequences for global electricity supply investment needs. *Energy*.

[112] Antwi M., Sedegah D. D. (2018). *Climate Change and Societal Change-Impact on Hydropower Energy Generation*.

[113] Yuebo X., Kabo-bah A. T., Kabo-Bah K. J., Domfeh M. K. (2018). *Hydropower Development-Review of the Successes and Failures in the World*.

[114] Onyutha C., Turyahabwe C., Kaweesa P. (2021). Impacts of climate variability and changing land use/land cover on river Mpanga flows in Uganda, east Africa. *Environmental Challenges*.

[115] Getirana A., Jung H. C., Van Den Hoek J., Ndehedehe C. E. (2020). Hydropower dam operation strongly controls Lake Victoria’s freshwater storage variability. *Science of the Total Environment*.

[116] Pervin L., Gan T. Y., Scheepers H., Islam M. S. (2021). Application of the HBV model for the future projections of water levels using dynamically downscaled global climate model data. *Journal of Water and Climate Change*.

[117] Pokhrel P., Ohgushi K., Fujita M. (2019). Impacts of future climate variability on hydrological processes in the upstream catchment of Kase river basin, Japan. *Applied Water Science*.

[118] ERA (2013). Developments and investment opportunities in renewable energy resources in Uganda. https://www.energyandminerals.go.ug.

[119] Get Fit Uganda (2019). *Annual Report 2019*.

[120] BMAU-MFPED (2017). *Water-Pumping Windmills in Karamoja: A Wasted Opportunity*.

[121] Ssenyimba S., Kiggundu N., Banadda N. (2020). Designing a solar and wind hybrid system for small-scale irrigation: a case study for Kalangala district in Uganda. *Energy, Sustainability and Society*.

[122] van Kooten G. C., Timilsina G. R. (2008). *Wind Power Development: Opportunities and Challenges*.

[123] Aarakit S. M., Ssennono V. F., Adaramola M. S. (2021). Estimating market potential for solar photovoltaic systems in Uganda. *Frontiers in Energy Research*.

[124] Kavuma C., Sandoval D., Dieu H. K. J. (2022). Analysis of solar photo-voltaic for grid integration viability in Uganda. *Energy Science & Engineering*.

[125] Get Fit Uganda (2020). *Annual Report 2020*.

[126] Mugagga R. G., Chamdimba H. B. N., Chamdimba N. (2019). A comprehensive review on status of solar PV growth in Uganda. *Journal of Energy Research and Reviews*.

[127] Avellino O. W. K., Mwarania F., Wahab A. H. A., Aime K. T., Aime K. T. (2018). Uganda solar energy utilization: current status and future trends. *International Journal of Scientific and Research Publications (IJSRP)*.

[128] Mukisa N., Zamora R., Lie T. T. (2020). Assessment of community sustainable livelihoods capitals for the implementation of alternative energy technologies in Uganda-Africa. *Renewable Energy*.

[129] van Hove E., Johnson N. G. (2021). Refugee settlements in transition: energy access and development challenges in northern Uganda. *Energy Research & Social Science*.

[130] Oloya I. T., Gutu T. J. L., Adaramola M. S. (2021). Techno-economic assessment of 10 MW centralised grid-tied solar photovoltaic system in Uganda. *Case Studies in Thermal Engineering*.

[131] Groenewoudt A. C., Romijn H. A., Alkemade F. (2020). From fake solar to full service: an empirical analysis of the solar home systems market in Uganda. *Energy for Sustainable Development*.

[132] Aarakit S. M., Ntayi J. M., Wasswa F., Adaramola M. S., Ssennono V. F. (2021). Adoption of solar photovoltaic systems in households: evidence from Uganda. *Journal of Cleaner Production*.

[133] Thadani H. L., Go Y. I. (2021). Integration of solar energy into low-cost housing for sustainable development: case study in developing countries. *Heliyon*.

[134] Mukisa N., Zamora R., Lie T. T. (2019). Feasibility assessment of grid-tied rooftop solar photovoltaic systems for industrial sector application in Uganda. *Sustainable Energy Technologies and Assessments*.

[135] Bhamidipati P. L., Elmer Hansen U., Haselip J. (2019). Agency in transition: the role of transnational actors in the development of the off-grid solar PV regime in Uganda. *Environmental Innovation and Societal Transitions*.

[136] Eder J. M., Mutsaerts C. F., Sriwannawit P. (2015). Mini-grids and renewable energy in rural Africa: how diffusion theory explains adoption of electricity in Uganda. *Energy Research & Social Science*.

[137] Farfan J., Breyer C. (2018). Combining floating solar photovoltaic power plants and hydropower reservoirs: a virtual battery of great global potential. *Energy Procedia*.

[138] Bambokela J. E., Belaid M., Muzenda E., Nhubu T. (2022). Developing a pilot biogas-solar PV system for farming communities in Botswana: case of Palapye. *Procedia Computer Science*.

[139] Puglia G., Moroni M., Fagnani R., Comodi G. (2017). A design approach of off-grid hybrid electric microgrids in isolated villages: a case study in Uganda. *Energy Procedia*.

[140] Mohammed S., Fatumah N., Shadia N. (2020). Drying performance and economic analysis of novel hybrid passive-mode and active-mode solar dryers for drying fruits in east Africa. *Journal of Stored Products Research*.

[141] Pandyaswargo A. H., Wibowo A. D., Onoda H. (2022). Socio-techno-economic assessment to design an appropriate renewable energy system for remote agricultural communities in developing countries. *Sustainable Production and Consumption*.

[142] Trotter P. A., Cooper N. J., Wilson P. R. (2019). A multi-criteria, long-term energy planning optimisation model with integrated on-grid and off-grid electrification—the case of Uganda. *Applied Energy*.

[143] (2013). Average year of growth for geothermal but vast potential. *Renewable Energy Focus*.

[144] Gude V. G. (2016). Geothermal source potential for water desalination—current status and future perspective. *Renewable and Sustainable Energy Reviews*.

[145] Janczik S., Kaltschmitt M. (2015). After a major dive, geothermal power is growing rapidly. *Renewable Energy Focus*.

[146] Lund J. W., Toth A. N. (2021). Direct utilization of geothermal energy 2020 worldwide review. *Geothermics*.

[147] Macgregor D. S. (2020). Regional variations in geothermal gradient and heat flow across the African plate. *Journal of African Earth Sciences*.

[148] Mutumba G., Adaramola M. S. (2021). Prospects and challenges of geothermal energy in Uganda prospects and challenges of geothermal energy in Uganda. *Journal of Energy Research and Reviews*.

[149] Zakkour P., Counts C. (2016). Formulating a geothermal energy policy, legal and regulatory framework for Uganda. https://www.researchgate.net/publication/310457844_FORMULATING_A_GEOTHERMAL_ENERGY_POLICY_LEGAL_AND_REGULATORY_FRAMEWORK_FOR_UGANDA.

[150] Carrara S. (2020). Reactor ageing and phase-out policies: global and regional prospects for nuclear power generation. *Energy Policy*.

[151] Kessides I. N. (2012). The future of the nuclear industry reconsidered: risks, uncertainties, and continued promise. *Energy Policy*.

[152] Kessides I. N. (2014). Powering Africa’s sustainable development: the potential role of nuclear energy. *Energy Policy*.

[153] Budnitz R. J., Rogner H. H., Shihab-eldin A. (2018). Expansion of nuclear power technology to new countries—SMRs, safety culture issues, and the need for an improved international safety regime. *Energy Policy*.

[154] Ramana M., Agyapong P. (2016). Thinking big? Ghana, small reactors, and nuclear power. *Energy Research & Social Science*.

[155] Postar S. (2017). The half-lives of African uranium: a historical review. *The Extractive Industries and Society*.

[156] Sah A., Lovering J., Maseli O., Saxena A. (2018). *Atoms for Africa: Is There a Future for Civil Nuclear Energy in Sub-Saharan Africa?*.

[157] Ferguson C. D. (2007). *Nuclear Energy: Balancing Benefits and Risks*.

[158] IAEA (2006). *Basic Infrastructure for a Nuclear Power Project*.

[159] International Energy Agency (2015). *Technology Roadmap: Nuclear Energy*.

[160] Musyoka D., Field R. M. (2018). Review of the environmental oversight framework in Kenya, in light of a nuclear power programme. *Progress in Nuclear Energy*.

[161] Adams S., Odonkor S. (2021). Status, opportunities, and challenges of nuclear power development in sub-Saharan Africa: the case of Ghana. *Progress in Nuclear Energy*.

[162] Ansah M. N. S., Agyekum E. B., Amoah P. A., Afornu B. K. (2021). Atoms for electricity generation in Africa: analysis of factors affecting the continent’s readiness. *Progress in Nuclear Energy*.

[163] Uhunamure S. E., Agyekum E. B., Durowoju O. S. (2021). Appraisal of nuclear energy as an alternative option in South Africa’s energy scenario: a multicriteria analysis. *Applied Sciences*.

[164] World Energy Council (2013). *World Energy Resources: Peat*.

[165] Hakizimana J. d. D. K., Yoon S. P., Kang T. J., Kim H. T., Jeon Y. S., Choi Y. C. (2016). Potential for peat-to-power usage in Rwanda and associated implications. *Energy Strategy Reviews*.

[166] Joosten H., Tapio-Biström M.-L., Toi S. (2012). *Peatlands—Guidance for Climate Change Mitigation through Conservation, Rehabilitation and Sustainable Use*.

[167] Mokveld K., von Eije S. (2018). Final energy report Uganda. https://www.rvo.nl/sites/default/files/2019/02/Final-Energy-report-Uganda.pdf.

[168] Jingchao Z., Kotani K., Saijo T. (2019). Low-quality or high-quality coal? Household energy choice in rural Beijing. *Energy Economics*.

[169] Popa A. (2016). *The Challenges of the U.S. Coal Industry and Lessons for Europe*.

[170] Miller B. G. (2011). The effect of coal usage on human health and the environment. *Clean Coal Engineering Technology*.

[171] Stanton T. (2013). *Understanding Coal’s Challenges and Recommended Regulatory Responses*.

[172] Carbon Fuels and Global Warming (2009). *Ten Problems with Coal*.

[173] Xu G., Wang X., Wang R., Yano G., Zou R. (2021). Anti-corruption, safety compliance and coal mine deaths: evidence from China. *Journal of Economic Behavior & Organization*.

[174] Sampedro J., Cui R. Y., McJeon H. (2021). Quantifying the reductions in mortality from air-pollution by cancelling new coal power plants. *Energy and Climate Change*.

[175] Mihalyi D., Scurfield T. (2021). How Africa’s prospective petroleum producers fell victim to the presource curse. *The Extractive Industries and Society*.

[176] Patey L. (2020). Oil, risk, and regional politics in east Africa. *The Extractive Industries and Society*.

[177] Olanya D. R. (2015). Will Uganda succumb to the resource curse? Critical reflections. *The Extractive Industries and Society*.

[178] Ovadia J. S. (2016). Local content policies and petro-development in sub-Saharan Africa: a comparative analysis. *Resources Policy*.

[179] Holterman D. (2014). The biopolitical war for life: extractivism and the Ugandan oil state. *The Extractive Industries and Society*.

[180] MEMD (2019). *The Oil and Gas Sector in Uganda: Frequently Asked Questions*.

[181] Garland C., Jagoe K., Wasirwa E. (2015). Impacts of household energy programs on fuel consumption in Benin, Uganda, and India. *Energy for Sustainable Development*.

[182] Matthews W. G. (2014). Opportunities and challenges for petroleum and LPG markets in sub-Saharan Africa. *Energy Policy*.

[183] Van Alstine J., Manyindo J., Smith L., Dixon J., Amanigaruhanga I. (2014). Resource governance dynamics: the challenge of “new oil” in Uganda. *Resources Policy*.

[184] Ogwang T., Vanclay F. (2021). Cut-off and forgotten?: livelihood disruption, social impacts and food insecurity arising from the east African crude oil pipeline. *Energy Research & Social Science*.

[185] Byakagaba P., Mugagga F., Nnakayima D. (2019). The socio-economic and environmental implications of oil and gas exploration: perspectives at the micro level in the Albertine region of Uganda. *The Extractive Industries and Society*.

[186] Ogwang T., Vanclay F., van den Assem A. (2018). Impacts of the oil boom on the lives of people living in the Albertine Graben region of Uganda. *The Extractive Industries and Society*.

[187] Barlow A. (2020). The politics of the temporary: Tanzanian local content in the east African crude oil pipeline. *The Extractive Industries and Society*.

[188] Harris A. S., Sigman R., Meyer-Sahling J. H., Mikkelsen K. S., Schuster C. (2020). Oiling the bureaucracy? Political spending, bureaucrats and the resource curse. *World Development*.

[189] Mawejje J. (2019). The oil discovery in Uganda’s Albertine region: local expectations, involvement, and impacts. *The Extractive Industries and Society*.

[190] Oppong N., Patey L., Soares de Oliveira R. (2020). Governing African oil and gas: boom-era political and institutional innovation. *The Extractive Industries and Society*.

[191] Omol D. K., Acaye O., Okot D. F., Bongomin O. (2020). Production of fuel oil from municipal plastic wastes using thermal and catalytic pyrolysis. *Journal of Energy Research and Reviews*.

[192] Abigaba M. L., Bengtsson J., Rosendahl K. E. (2021). How valuable is the option to defer Uganda’s crude oil production?. *Scientific African*.

[193] Kinyera P. B. (2019). Land, oil and expressions of citizenship in Uganda’s Albertine graben. *The Extractive Industries and Society*.

[194] Xu W., Zhang X., Shang F., Fang L., Liu J., Yang X. (2018). An integrated quantitative approach for determination of net reservoir cutoffs: a case study of Q oil field, Lake Albert, Uganda. *Journal of African Earth Sciences*.

[195] Bamwesigye D., Kupec P., Chekuimo G. (2020). Charcoal and wood biomass utilization in Uganda: the socioeconomic and environmental dynamics and implications. *Sustainability*.

[196] To L. S., Seebaluck V., Leach M. (2018). Future energy transitions for bagasse cogeneration: lessons from multi-level and policy innovations in Mauritius. *Energy Research & Social Science*.

[197] Kasinath A., Fudala-Ksiazek S., Szopinska M. (2021). Biomass in biogas production: pretreatment and codigestion. *Renewable and Sustainable Energy Reviews*.

[198] Wasajja H., Lindeboom R. E. F., Van Lier J. B., Aravind P. (2020). Techno-economic review of biogas cleaning technologies for small scale off- grid solid oxide fuel cell applications. *Fuel Processing Technology*.

[199] Buchholz T., Da Silva I., Furtado J. (2012). Power from wood gasifiers in Uganda: a 250 kW and 10 kW case study. *Proceedings of the Institution of Civil Engineers-Energy*.

[200] Nakamya M., Romstad E. (2020). Ethanol for an agriculture-based developing economy: a computable general equilibrium assessment for Uganda. *Energy for Sustainable Development*.

[201] Namugenyi I., Coenen L., Scholderer J. (2022). Realising the transition to bioenergy: integrating entrepreneurial business models into the biogas socio-technical system in Uganda. *Journal of Cleaner Production*.

[202] Clemens H., Bailis R., Nyambane A., Ndung’u V. (2018). Africa biogas partnership program: a review of clean cooking implementation through market development in east Africa. *Energy for Sustainable Development*.

[203] Tumusiime E., Kirabira J. B., Musinguzi W. B. (2019). Long-life performance of biogas systems for productive applications: the role of R & D and policy. *Energy Reports*.

[204] Ogwang I., Kasedde H., Nabuuma B., Kirabira J. B., Lwanyaga J. D. (2021). Characterization of biogas digestate for solid biofuel production in Uganda. *Scientific African*.

[205] Nalunga A., Mugisha J., Walekhwa P., Smith J. (2019). The dynamics of Household labor allocation to biogas production, farm and non-farm activities in central Uganda. *Renewable Energy*.

[206] Lwiza F., Mugisha J., Walekhwa P. N., Smith J., Balana B. (2017). Dis-adoption of household biogas technologies in central Uganda. *Energy for Sustainable Development*.

[207] Walekhwa P. N., Lars D., Mugisha J. (2014). Economic viability of biogas energy production from family-sized digesters in Uganda. *Biomass and Bioenergy*.

[208] Kabyanga M., Balana B. B., Mugisha J., Walekhwa P. N., Smith J., Glenk K. (2018). Are smallholder farmers willing to pay for a flexible balloon biogas digester? Evidence from a case study in Uganda. *Energy for Sustainable Development*.

[209] Ijoma G. N., Mutungwazi A., Mannie T., Nurmahomed W., Matambo S., Hildebrandt D. (2022). Addressing the water-energy nexus: a focus on the barriers and potentials of harnessing wastewater treatment processes for biogas production in sub Saharan Africa. *Heliyon*.

[210] Nevzorova T., Kutcherov V. (2019). Barriers to the wider implementation of biogas as a source of energy: a state-of-the-art review. *Energy Strategy Reviews*.

[211] Wardle J. M., Fischer A., Tesfaye Y., Smith J. (2021). Seasonal variability of resources: the unexplored adversary of biogas use in rural Ethiopia. *Current Research in Environmental Sustainability*.

[212] Mukeshimana M. C., Zhao Z. Y., Ahmad M., Irfan M. (2021). Analysis on barriers to biogas dissemination in Rwanda: AHP approach. *Renewable Energy*.

[213] Patinvoh R. J., Taherzadeh M. J. (2019). Challenges of biogas implementation in developing countries. *Current Opinion in Environmental Science & Health*.

[214] Roopnarain A., Adeleke R. (2017). Current status, hurdles and future prospects of biogas digestion technology in Africa. *Renewable and Sustainable Energy Reviews*.

[215] Surendra K. C., Takara D., Hashimoto A. G., Khanal S. K. (2014). Biogas as a sustainable energy source for developing countries: opportunities and challenges. *Renewable and Sustainable Energy Reviews*.

[216] Musinguzi W. B., Okure M. A. E., Wang L., Sebbit A., Løvås T. (2012). Thermal characterization of Uganda’s *Acacia hockii*, *Combretum molle*, *Eucalyptus grandis* and *Terminalia glaucescens* for gasification. *Biomass and Bioenergy*.

[217] Janajreh I., Adeyemi I., Raza S. S., Ghenai C. (2021). A review of recent developments and future prospects in gasification systems and their modeling. *Renewable and Sustainable Energy Reviews*.

[218] Olupot P. W., Candia A., Menya E., Walozi R. (2016). Characterization of rice husk varieties in Uganda for biofuels and their techno-economic feasibility in gasification. *Chemical Engineering Research and Design*.

[219] Yusuf A. A., Inambao F. L. (2019). Bioethanol production from different Matooke peels species: a surprising source for alternative fuel. *Case Studies in Thermal Engineering*.

[220] S Mutumba G., Mubiinzi G., Milly K., Otim J. (2022). Renewable energy consumption and economic growth in Uganda. *Journal of Energy Research and Reviews*.

[221] Twinomuhangi R., Martin Kato A., Sebbit A. M. (2021). The energy and climate change nexus in Uganda: policy challenges and opportunities for climate compatible development. *Global Warming and Climate Change*.

[222] Thapa S., Morrison M., Parton K. A. (2021). Willingness to pay for domestic biogas plants and distributing carbon revenues to influence their purchase: a case study in Nepal. *Energy Policy*.

[223] Ali M. M., Ndongo M., Bilal B., Yetilmezsoy K., Youm I., Bahramian M. (2020). Mapping of biogas production potential from livestock manures and slaughterhouse waste: a case study for African countries. *Journal of Cleaner Production*.

[224] Daniyan I. A., Daniyan O. L., Abiona O. H., Mpofu K. (2019). Development and optimization of a smart system for the production of biogas using poultry and pig dung. *Procedia Manufacturing*.

[225] Amulen J., Kasedde H., Serugunda J., Lwanyaga J. D. (2022). Energy conversion and management: X the potential of energy recovery from municipal solid waste in Kampala city, Uganda by incineration internal rate of return. *Energy Conversion and Management X*.

[226] Bongomin O., Nziu P. (2022). A critical review on the development and utilization of energy systems in Uganda. https://www.preprints.org/manuscript/202202.0361/v1.

